# Exploring architectures displaying multimeric presentations of a trihydroxypiperidine iminosugar

**DOI:** 10.3762/bjoc.11.282

**Published:** 2015-12-16

**Authors:** Camilla Matassini, Stefania Mirabella, Andrea Goti, Inmaculada Robina, Antonio J Moreno-Vargas, Francesca Cardona

**Affiliations:** 1 Dipartimento di Chimica “Ugo Schiff”, Università di Firenze, Via della Lastruccia 3-13, 50019 Sesto Fiorentino (FI), Italy; 2 Departamento de Química Orgánica, Facultad de Química, Universidad de Sevilla, c/ Prof. García González 1, E-41012, Sevilla, Spain

**Keywords:** dendrimers, glycosidase inhibitors, iminosugars, multivalency, piperidine alkaloids

## Abstract

The synthesis of new multivalent architectures based on a trihydroxypiperidine α-fucosidase inhibitor is reported herein. Tetravalent and nonavalent dendrimers were obtained by means of the click chemistry approach involving the copper azide-alkyne-catalyzed cycloaddition (CuAAC) between suitable scaffolds bearing terminal alkyne moieties and an azido-functionalized piperidine as the bioactive moiety. A preliminary biological investigation is also reported towards commercially available and human glycosidases.

## Introduction

Iminosugars are well-known naturally occurring glycomimetics with a nitrogen atom replacing the endocyclic oxygen, mainly recognized as inhibitors of carbohydrate-processing enzymes (glycosidases) [1–2]. In quite sharp contrast the multivalent effect, widely investigated in the field of carbohydrate–lectin interactions [3], has remained essentially unexplored concerning glycosidase inhibition up to 2010. Indeed, the first examples of multivalent iminosugars gave disappointing results in terms of inhibition and therefore were not encouraging [4–6]. However, following the first promising affinity enhancements reported towards jack-bean α-mannosidase for a trivalent derivative of 1-deoxynojirimycin [7], over the past six years the multivalent effect in glycosidase inhibition has received an increasing attention by the scientific community, rapidly emerging as a hot topic in glycoscience. Three excellent recent reviews collect the efforts of the researchers both in the synthesis and in the biological evaluation of the new multivalent structures [8–10]. In particular, remarkable high multivalent effects towards jack-bean α-mannosidase were reported for fullerene- [11], cyclodextrin- [12–13] and porphyrin- [14] based scaffolds decorated with 1-deoxynojirimycin (DNJ) or 1-deoxymannojirimycin as the bioactive iminosugars. Self-assembled DNJ-based glycopeptides also experienced a remarkable multivalent effect towards jack bean α-mannosidase [15].

Some mechanisms of action have been also proposed based on the efforts recently devoted to understand the multivalent glycosidase inhibition interactions [10]. Furthermore, some applications of these multivalent systems have been reported involving other glycosidases of therapeutic interest, in particular in the field of rare genetic diseases connected to misfolded proteins [16–18].

While deoxynojrimycin (DNJ) is commonly employed to build diversified multivalent architectures, relative few examples have been reported with different bioactive molecules (namely pyrrolidine- and pyrrolizidine-based iminosugars) [19–21].

Moreover, trivalent pyrrolidine derivatives have been recently employed to probe the multivalent effect towards α-L-fucosidase inhibition [22], which may be clinically relevant in the treatment of fucosidosis metabolic disorder and *Helicobacter pylori* infection, as well as in the elucidation of the biological role of α-L-fucosidase in spermiogenesis and sperm maturation [23].

**Scheme 1:**
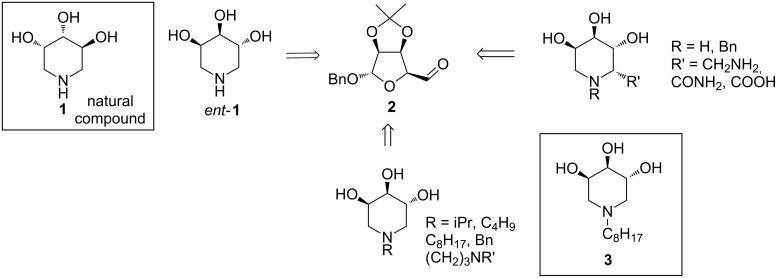
Double reductive amination on aldehyde **2** allowed the synthesis of trihydroxypiperidines, among which the enantiomer of natural compound **1** and the *N*-alkylated piperidine **3**.

Following our interest in the synthesis of natural alkaloids and their unnatural analogs we recently developed a straightforward synthetic strategy for the synthesis of diversely functionalized trihydroxypiperidines through double reductive amination of the D-mannose-derived aldehyde **2** (Scheme 1) [24–25].

Among the 1-azasugars accessed with this methodology, our attention was drawn to the enantiomer of natural 3,4,5-trihydroxypiperidine (**1**), *ent*-**1**, which showed good inhibitory activity and excellent selectivity towards commercial bovine kidney [25–26] and other mammalian [27] α-L-fucosidases. Moreover, we recently found that *N*-alkylated trihydroxypiperidine **3** showed some chaperoning activity once incubated with human fibroblasts derived from Gaucher bearing N370/RecNcil mutations, being able to rescue the residual enzyme activity up to 1.25 fold [28].

These findings, together with the opportunity to easily functionalize the trihydroxypiperidine skeleton with an azido moiety at the terminal *N*-alkyl chain, prompted us to investigate the multimerization of compound *ent*-**1** with the aim of studying its inhibitory activity when the molecule decorates a multivalent scaffold. Herein we report the synthesis of a tetra- and a nonavalent polyhydroxypiperidine iminosugar, by exploiting the Cu^I^-catalyzed azide-alkyne cycloadditions (CuAAC) [29–32] with two different dendrimeric alkyne scaffolds.

## Results and Discussion

The “masked” dialdehyde intermediate **2** was easily synthesized in four steps and 80% overall yield from D-mannose without the need of any chromatographic purification, by following a slight modification of the published procedure [24,33]. The versatility of our synthetic methodology allows access to differently substituted *N*-alkylated trihydroxypiperidines by simply using the same aldehyde and different amines as the nitrogen source in a double reductive amination strategy [24–25].

**Scheme 2:**
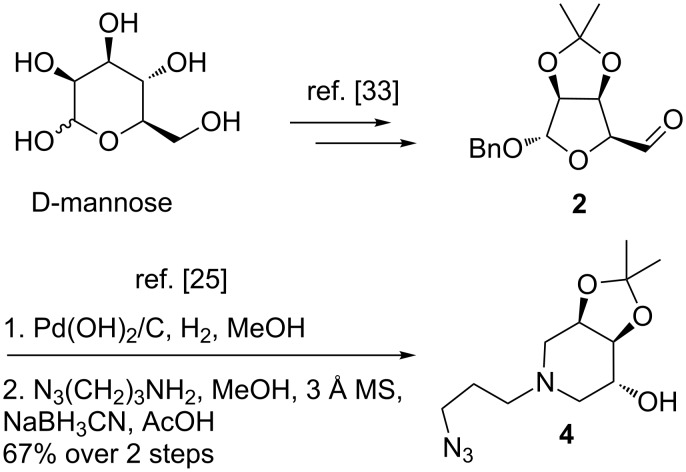
Synthesis of key azide intermediate **4** through the double reductive amination strategy from “masked” dialdehyde intermediate **2**.

In particular, catalytic hydrogenation with Pd(OH)_2_/C in MeOH followed by reductive amination of the formed dialdehyde intermediate with 3-azidopropyl-1-amine [34] in the presence of NaBH_3_CN and AcOH allowed access to *N*-alkylated piperidine **4** in 67% yield (Scheme 2) [25].

**Scheme 3:**
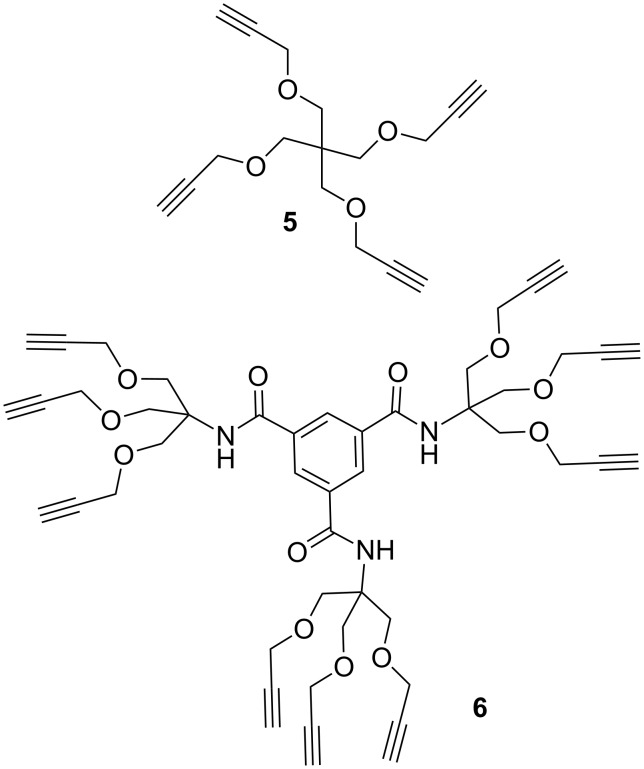
Tetravalent and nonavalent alkyne scaffolds.

With the key azido intermediate **4** in hands, we proceeded with the synthesis of two different scaffolds bearing terminal alkyne moieties suitable for conjugation to compound **4**. A preliminary evaluation of the role of the valency in enhancing the inhibitory activity of the iminosugar was investigated by synthesizing a tetravalent and a nonavalent scaffold. The tetravalent scaffold **5** (Scheme 3) was obtained by propargylation of pentaerythritol with propargyl bromide and NaH following a previously published procedure [35], while the dendrimeric nonavalent scaffold **6** (Scheme 3) was obtained in good yield from the reaction of tris[(propargyloxy)methyl]aminomethane with trimesoyl chloride, as we recently reported [21].

**Scheme 4:**
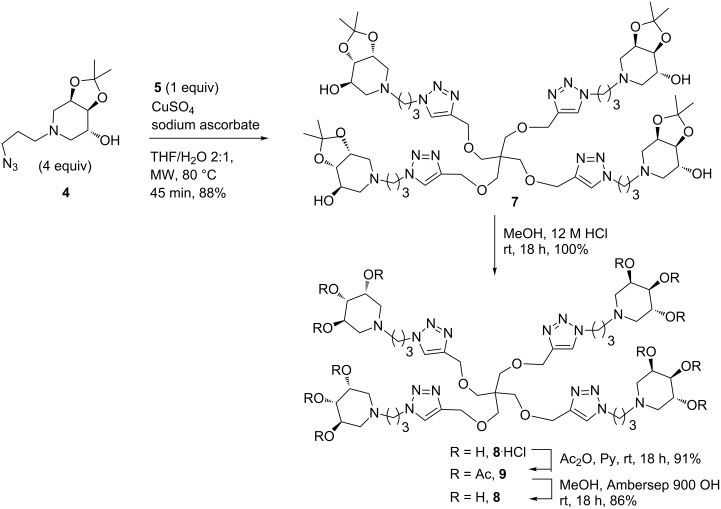
Synthesis of the tetravalent adduct **7** by CuAAC reaction and its deprotection/purification process to obtain the final compound **8**.

The CuAAC reaction of the azido derivative **4** (4.0 equivalents) with scaffold **5** was performed with CuSO_4_/sodium ascorbate in THF/H_2_O 2:1 in a MW reactor at 80 °C for 45 minutes, affording the expected tetravalent iminosugar derivative **7** in 88% yield after flash column chromatography (Scheme 4).

Subsequent treatment of **7** in acidic MeOH at room temperature for 18 hours gave the hydrochloride salt **8** (Scheme 4), which was passed through ion exchange resin Dowex 50WX8-200 eluting successively with MeOH, H_2_O and 6% NH_4_OH. This general purification procedure, successfully employed by us for several deprotected monovalent compounds [25], resulted much less efficient with the more basic and hydrophilic adduct **8**. Since in this case most of the compound was recovered in the first fraction with MeOH as hydrochloride salt and only a small amount in the NH_4_OH final fraction as free amine, we were forced to turn to a protection/deprotection methodology. Hence, the methanolic fraction was acetylated by treatment with excess of pyridine and acetic anhydride, affording compound **9** with 91% yield after flash column chromatography. After treatment with strongly basic resin Ambersep 900-OH, compound **8** was obtained pure in 86% yield (Scheme 4). This compound and the free amine previously recovered by DOWEX elution of **8**^.^HCl with 6% NH_4_OH were proved to be identical by ^1^H NMR analysis.

**Scheme 5:**
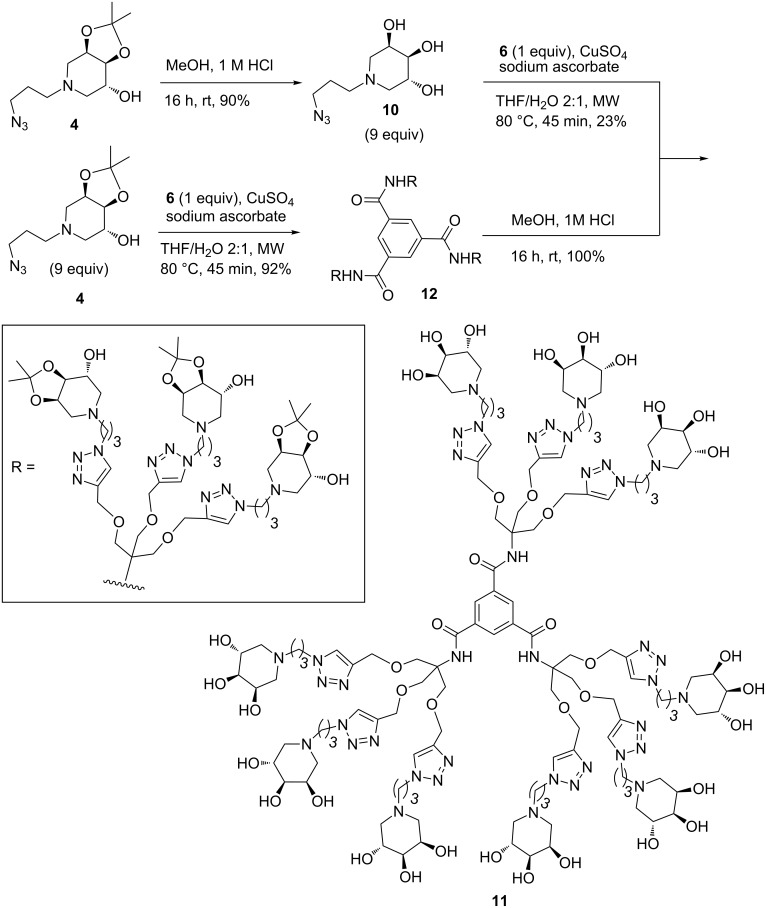
Synthesis of nonavalent adduct **11** by CuAAC reaction and its deprotection.

To address the synthesis of the nonavalent compound avoiding purification problems we first tried the deprotection of the acetonide groups prior to CuAAC reaction. Compound **4** was treated with 1 M HCl in MeOH at room temperature for 16 hours and then passed through Dowex 50WX8-200, following the general purification procedure previously described, to obtain the polyhydroxylated azido derivative **10** [25] in 90% yield. The CuAAC reaction of compound **10** (9 equiv) with the nonavalent alkyne scaffold **6**, performed with CuSO_4_/sodium ascorbate in THF/H_2_O 2:1 heating in a MW reactor at 80 °C for 90 minutes, gave the nonavalent compound **11** in 23% yield, after flash column chromatography (Scheme 5). The low yield observed for the click reaction of the deprotected azido derivative **10** can be ascribed to the tricky purification of **11** that was recovered from silica gel only by eluting with 33% NH_4_OH due to its high basicity (see Experimental section).

For this reason we decided to change the strategy by performing the click reaction on the protected azido derivative **4**, analogously to what reported with the tetravalent scaffold **5**. Reaction of **4** (9 equiv) with the nonavalent alkyne scaffold **6** gave compound **12** in excellent 92% yield (Scheme 5). After treatment in acidic methanol at room temperature for 16 h, the hydrochloride salt of the nonavalent adduct **11** was obtained in quantitative yield. However, due to the purification problems previously encountered for free amine **11**, we decided to purify the nonavalent derivative **11**·HCl by size exclusion chromatography. Indeed, in this case, the use of the strongly basic resin Ambersep 900-OH is hampered by the presence of the amidic bonds in compound **11**, which are not stable to strong basic conditions. Therefore, **11**·HCl was quickly passed over Sephadex LH-20 resin, eluted with water, to obtain the purified compound as hydrochloride salt, which was thus employed in further biological evaluation.

**Scheme 6:**
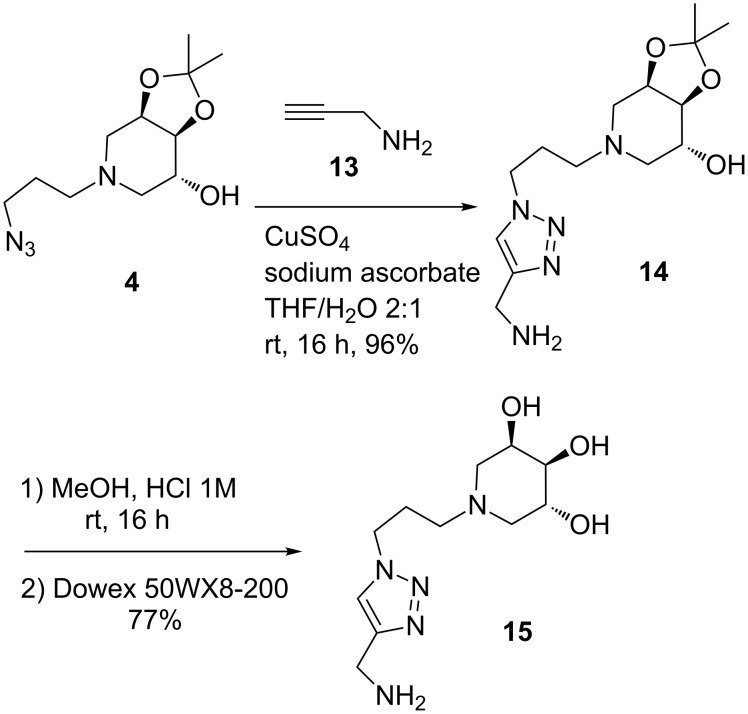
Synthesis of the monovalent iminosugar **15** by CuAAC reaction and subsequent deprotection of the hydroxy groups.

In order to evaluate the relative inhibitory activity enhancement of these new multimeric systems, a proper monovalent counterpart was also synthesized. In particular, starting from azidopiperidine **4**, the CuAAC reaction was performed with propargylamine (**13**) in the presence of CuSO_4_/sodium ascorbate in THF/H_2_O 2:1 at room temperature affording adduct **14** in 96% yield (Scheme 6). Final deprotection by treatment with MeOH/HCl and eluting over ion exchange resin Dowex 50WX8-200, afforded the monovalent compound **15** in 77% yield.

Searching for a multivalent effect towards α-fucosidase inhibition, the tetravalent compound **8**, the nonavalent compound **11^.^**HCl, as well as the monovalent **15**, were assayed as glycosidase inhibitors towards α-fucosidase (EC 3.2.1.51) from bovine kidney. Eight further commercially available glycosidases were also considered in order to investigate a possible change in selectivity due to the polyhydroxypiperidine multimerization. The results are summarized in Table 1, where inhibition of simple 3,4,5-trihydroxypiperidine *ent*-**1** is also reported for sake of completeness.

**Table 1:** Inhibitory activity of compounds *ent*-**1**, **8**, **11^.^**HCl, **15** towards glycosidases. Percentages of inhibition at 1mM of inhibitor (IC_50_ in parentheses [µM]) were reported.

Commercially available glycosidases	Evaluated compounds			

	*ent*-**1**	**8**	**11**·HCl	**15**

**α-L-fucosidase** bovine kidney	89 [25] (90.3)	45	41	28
**α-galactosidase** coffee beans	–	39	58	–
**β-galactosidase** *Escherichia coli* *Aspergillus oryzae*	–	–	–	–
**α-glucosidase** *Saccharomyces cerevisiae* rice	–	29	–	–
**amyloglucosidase** *Aspergillus niger*	–	57	86 (179)	45 (1100)
**β-glucosidase** almonds	–	–	–	–
**α-mannosidase** jack beans	–	–	32	–
**β-mannosidase** snail	–	–	–	–
**β-*N*-acetylglucosaminidase** jack beans	–	–	–	–

– : no inhibition was detected at 1 mM concentration of the corresponding compound.

Unfortunately, concerning the α-fucosidase inhibition, the multimerization of active polyhydroxypiperidine *ent*-**1** onto both tetravalent and nonavalent scaffolds (**8** and **11**·HCl), as well as the simple functionalization at the nitrogen atom (**15**) led to a dramatic decrease of its inhibitory activity, demonstrating a low tendency of this enzyme to be influenced by multivalent presentation [22]. Conversely, some effect in the change of selectivity can be observed as attested by the increase on amyloglucosidase inhibition. In this case, while *ent*-**1** does not inhibit this enzyme at all, increasing inhibitory activity was observed passing from its tetravalent derivative (57%) to the nonavalent (86%) one. For this latter compound (**11**·HCl), IC_50_ = 179 μM was calculated [36]. This outcome is particularly desirable with multimeric iminosugars for increasing the overall binding affinity of weak inhibitors, as recently pointed out by Winum and Ulrich in a recent review [37]. It should also be noted that analogous changes in selectivity towards different glycosidases comparing monovalent with multivalent polymeric iminosugars were recently documented [38].

However, by comparing the IC_50_ value of **11**·HCl with the monovalent counterpart **15** (relative inhibition potency = 6), an effective multivalent effect cannot be claimed, since the enhancement observed is not higher than 9-fold (the valency of the dendrimeric compound).

Since in our experience the inhibition data towards commercial and human glycosidases do not always match [28], we then decided to evaluate the most promising nonavalent compound **11**·HCl also towards human glycosidases. In particular, compound **11**·HCl was incubated at 1 mM in an extract from a pool of leukocytes isolated from healthy donors and the α-fucosidase activity was estimated by fluorescence measurements. A 46% inhibitory activity, calculated with respect to the blank control, was found: this data is in complete agreement with the value obtained towards the commercial glycosidase. We finally evaluated **11**·HCl against two lysosomal enzymes, *N*-acetylgalactosamine-6-sulfate sulfatase (GALNS) and iduronate 2-sulfatase that, being dimers [39], are in principle more prone to accept multivalent substrates. Actually only low inhibition activities (50% and 69%, respectively) were measured for these two enzymes, that represent appealing targets for the treatment of rare metabolic disorders such as mucopolysaccharidosis type IVA (Morquio A) and mucopolysaccharidosis type II (Hunter syndrome).

## Conclusion

In conclusion, the synthesis of two new dendrimeric iminosugars (namely a tetravalent and a nonavalent) based on a trihydroxypiperidine α-fucosidase inhibitor was achieved in this work. Based on a key trihydroxypiperidine intermediate bearing a terminal azido moiety, the CuAAC approach was investigated either on the protected and deprotected iminosugar, with different purification techniques employed in both cases. A monomer reference compound was also synthesized for comparison. Biological evaluation against a panel of eleven commercially available glycosidases showed that fucosidase inhibition is lost, while an unexpected amyloglucosidase inhibition is observed with these dendrimeric compounds. Moreover, preliminary evaluation towards human glycosidases showed moderate inhibition towards particularly relevant enzymes, so there is clearly space to consider expansion of our current amyloglucosidase inhibitors so as to generate therapeutically significant and specific inhibitors.

## Experimental

**General methods:** Commercial reagents were used as received. All reactions were carried out under magnetic stirring and monitored by TLC on 0.25 mm silica gel plates (Merck F_254_). Column chromatographies were carried out on Silica Gel 60 (32–63 μm) or on silica gel (230–400 mesh, Merck). Yields refer to spectroscopically and analytically pure compounds unless otherwise stated. ^1^H NMR spectra were recorded on a Varian Mercury-400 or on a Varian INOVA 400 instruments at 25 °C. ^13^C NMR spectra were recorded on a Varian Gemini-200 or on a Varian Gemini-300 spectrometer. Chemical shifts are reported relative to TMS (^1^H: δ = 0.00 ppm) and CDCl_3_ (^13^C: δ = 77.0 ppm). Integrals are in accordance with assignments, coupling constants are given in Hz. For detailed peak assignments 2D spectra were measured (COSY, HSQC, NOESY, and NOE as necessary). Small scale microwave-assisted syntheses were carried out in a microwave apparatus for synthesis (CEM Discover) with an open reaction vessel and external surface sensor. IR spectra were recorded with a BX FTIR Perkin-Elmer system spectrophotometer. ESIMS spectra were recorded with a Thermo Scientific™ LCQ fleet ion trap mass spectrometer. Elemental analyses were performed with a Perkin-Elmer 2400 analyzer. Optical rotation measurements were performed on a JASCO DIP-370 polarimeter.

**Protected tetravalent iminosugar 7:** In a similar manner as described in [21] to a solution of **4** (89 mg, 0.35 mmol) in 1.8 mL of a 2:1 THF/H_2_O mixture, CuSO_4_ (30 mol %, 3.8 mg, 0.024 mmol), sodium ascorbate (60 mol %, 9.5 mg, 0.048 mmol) and **5** (23 mg, 0.08 mmol) were added. The reaction mixture was heated in a MW reactor at 80 °C for 45 min, until TLC analysis showed the disappearance of the starting material. After filtration through Celite^®^, the solvent was removed under reduced pressure and the crude product was purified by gradient FCC (from CH_2_Cl_2_/MeOH 10:1 to CH_2_Cl_2_/MeOH/NH_4_OH 6% 10:1:0.1) affording pure **7** (92 mg, 0.07 mmol, 88%) as a pale yellow oil. *R*_f_ 0.32 (CH_2_Cl_2_/MeOH/NH_4_OH 6% 10:1:0.1); [α]_D_^24^ +4.88 (*c* 1.92, CHCl_3_); ^1^H NMR (400 MHz, CDCl_3_) δ 7.67 (s, 4H, H-triazole), 4.54 (s, 8H, O*CH_2_-*triazole), 4.44 (t, *J* = 6.6 Hz, 8H, 3’-H), 4.32 (dd, *J =* 11.7, 5.3 Hz, 4H, 3-H), 4.05 (t, *J* = 4.7 Hz, 4H, 4-H), 3.98–3.95 (m, 4H, 5-H), 3.42 (s, 8H, C*CH_2_*O), 2.71 (dd, *J* = 12.0, 5.3 Hz, 4H, 2-Ha), 2.59 (dd, *J* = 11.7, 2.9 Hz, 4H, 6-Ha), 2.47–2.41 (m, 8H, 2-Hb + 6-Hb), 2.36 (t, *J* = 6.6 Hz, 8H, 1’-H), 2.11–2.03 (m, 8H, 2’-H), 1.49 (s, 12H, Me), 1.35 (s, 12H, Me) ppm; ^13^C NMR (50 MHz, CDCl_3_) δ 145.1 (s, 4C, C-triazole), 123.3 (d, 4C, C-triazole), 109,0 (s, 4C, acetal), 77.3 (d, 4C, C-4), 72.2 (d, 4C, C-3), 68.9 (t, 4C, C*CH_2_*O), 68.0 (d, 4C, C-5), 64.8 (t, 4C, O*CH_2_*-triazole), 56.1 (t, 4C, C-6), 54.8 (t, 4C, C-2), 53.4 (t, 4C, C-1’), 47.7 (t, 4C, C-3’), 45.2 (s, *C*CH_2_O ), 28.3 (q, 4C, Me), 27.3 (t, 4C, C-2’), 26.3 (q, 4C, Me) ppm; MS (ESI) *m*/*z* (%): 1335.83 (100) [M + Na]^+^; IR (CDCl_3_): ν 3629, 3416, 3143, 2988, 2939, 2826, 2247, 1724, 1665, 1550, 1468, 1383, 1220, 1058 cm^−1^; anal. calcd for C_61_H_100_N_16_O_16_: C, 55.78; H, 7.67; N, 17.06; found: C, 55.74; H, 7.66; N, 16.83.

**Peracetylated tetravalent iminosugars 9:** In a similar manner as described in [21] to a solution of **7** (85 mg, 0.065 mmol) in 35 mL of methanol, 0.22 mL (10.0 equiv) of 37% HCl were added and the mixture was stirred at room temperature for 18 hours. After that a TLC analysis showed the disappearance of the starting material, the solvent was removed under reduced pressure. Successively elution with MeOH, H_2_O and 6% NH_4_OH over ion exchange resin Dowex 50WX8-200 afforded 9 mg (0.008 mmol) of corresponding free amine and 42 mg (0.032 mmol) of hydrochloride salt (MeOH fraction). This fraction was dissolved in pyridine (1.2 mL) and acetic anhydride (0.8 mL) was added. The solution was stirred at room temperature for 18 hours. Then, after concentration under reduced pressure, the crude mixture was purified by gradient FCC (from CH_2_Cl_2_/MeOH 20:1 to 5:1) affording pure **9** (48 mg, 0.029 mmol, 91%) as an oil. *R*_f_ 0.22 (CH_2_Cl_2_/MeOH 10:1); [α]_D_^22^ −42.1 (*c* 1.45, CHCl_3_); ^1^H NMR (400 MHz, CDCl_3_) δ 7.55 (s, 4H, H-triazole), 5.22 (dt, *J =* 5.4, 2.7 Hz, 4H, 3-H), 5.07 (dt, *J =* 8.2, 4.4 Hz, 4H, 5-H), 4.90 (dd, *J =* 8.8, 3.4 Hz, 4H, 4-H), 4.48 (s, 8H, O*CH_2_-*triazole), 4.42–4.29 (m, 8H, 3’-H), 3.39 (s, 8H, C*CH_2_*O), 2.92 (d, *J* = 8.3 Hz, 4H, 6-Ha), 2.77 (dd, *J* = 12.1, 3.9 Hz, 4H, 2-Ha), 2.40–2.33 (m, 12H, 2-Hb + 1’-H), 2.21–2.15 (m, 4H, 6-Hb), 2.03 (s, 12H, Ac), 2.01–1.93 (m, 8H, 2’-H), 1.99 (s, 12H, Ac), 1.97 (s, 12H, Ac) ppm; ^13^C NMR (50 MHz, CDCl_3_) δ 169.2 (s, 12C, OAc), 144.3 (s, 4C, C-triazole), 122.3 (d, 4C, C-triazole), 70.6 (d, 4C, C-4), 68.9 (t, 4C, C*CH_2_*O), 67.8 (d, 4C, C-5), 67.5 (d, 4C, C-3), 64.7 (t, 4C, O*CH_2_-*triazole), 53.6 (t, 4C, C-2), 53.2 (t, 4C, C-6), 53.0 (t, 4C, C-1’), 47.4 (t, 4C, C-3’), 45.3 (s, *C*CH_2_O ), 27.2 (t, 4C, C-2’), 20.8-20.5 (q, 12C, OAc) ppm; MS (ESI) *m*/*z* (%): 1679.92 (100) [M + Na]^+^; IR (CDCl_3_): ν 3451, 3145, 2960, 2874, 2825, 2258, 2246, 1743, 1663, 1470, 1437, 1372, 1231, 1049 cm^−1^; anal. calcd for C_73_H_108_N_16_O_28_: C, 52.89; H, 6.57; N, 13.52; found: C, 52.52; H, 6.25; N, 13.90.

**Polyhydroxylated tetravalent iminosugar 8:** In a similar manner as described in [21] a suspension of **9** (31 mg, 0.019 mmol) and ion exchange resin Ambersep-900 OH (500 mg) in 10 mL of methanol was slowly stirred at room temperature for 16 h. After filtration of resin on Celite^®^, the solvent was removed under reduced pressure affording pure **8** (19 mg, 0.016 mmol, 86% yield) as a waxy solid. [α]_D_^23^ −13.0 (*c* 1.1, H_2_O); ^1^H NMR (400 MHz, D_2_O) δ 7.83 (s, 4H, H-triazole), 4.38 (s, 8H, O*CH_2_*-triazole), 4.31 (t, *J* = 6.8 Hz, 8H, 3’-H), 3.86–3.82 (m, 4H, 3-H), 3.72 (td, *J* = 8.8, 4.4 Hz, 4H, 5-H), 3.58–3.34 (m, 4H, 4-H), 3.25 (s, 8H, C*CH_2_*O), 2.72–2.65 (m, 8H, 2-Ha + 6-Ha), 2.28–2.11 (m, 12H, 2-Hb + 1’-H), 2.00–1.9 (m, 12H, 6-Hb + 2’-H) ppm; ^13^C NMR (50 MHz, D_2_O) δ 143.2 (s, 4C, C-triazole), 124.0 (d, 4C, C-triazole), 72.8 (d, 4C, C-4), 67.1 (t, 4C, C*CH_2_*O), 66.8 (d, 4C, C-3), 66.6 (d, 4C, C-5), 62.6 (t, 4C, O*CH_2_-*triazole), 55.7 (t, 4C, C-6), 54.8 (t, 4C, C-2), 52.9 (t, 4C, C-1’), 47.7 (t, 4C, C-3’), 43.7 (s, *C*CH_2_O), 25.4 (t, 4C, C-2’) ppm; MS (ESI) *m*/*z* (%): 1175.79 (100) [M + Na]^+^; anal. calcd for C_49_H_84_N_16_O_16_: C 51.03, H 7.34, N 19.43; found: C, 50.71; H, 7.46; N, 19.62.

**Deprotected nonavalent iminosugar 11:** Analogously as described in [21] to a solution of **10** (50 mg, 0.23 mmol) in 3 mL of a 2:1 THF/H_2_O mixture, CuSO_4_ (30 mol %, 1.2 mg, 0.007 mmol), sodium ascorbate (80 mol %, 4.1 mg, 0.021 mmol) and **6** (21 mg, 0.025 mmol) were added. The reaction mixture was heated in a MW reactor at 80 °C for 90 min, until TLC analysis showed the disappearance of the nonavalent alkyne scaffold. After filtration through Celite^®^, the solvent was removed under reduced pressure and the crude product was purified by gradient FCC (from CH_2_Cl_2_/MeOH/NH_4_OH 6% 1:1:0.3 to NH_4_OH 33%) to afford pure **11** (16 mg, 0.006 mmol, 23%) as a pale yellow oil. *R*_f_ 0.19 (CH_2_Cl_2_/MeOH/NH_4_OH 33% 1:1:0.3); [α]_D_^21^ −21.31 (*c* 1.30, H_2_O); ^1^H NMR (400 MHz, D_2_O) δ 7.87 (s, 3H, Ar), 7.82 (s, 9H, H-triazole), 4.45 (s, 18H, O*CH_2_*-triazole), 4.22 (t, *J* = 6.7 Hz, 18H, 3’-H), 3.82–3.80 (m, 9H, 3-H), 3.71–3.66 (m, 27H, 5-H + O*CH_2_*CNH), 3.33–3.29 (m, 9H, 4-H), 2.72 (d, *J* = 9.9 Hz, 9H, 6-Ha), 2.66 (d, *J* = 10.3 Hz, 9H, 2-Ha), 2.28–2.23 (m, 18H, 1’-H), 2.17 (d, *J* = 10.2 Hz, 9H, 6-Hb), 1.96–1.84 (m, 27H, 2-Hb + 2’-H) ppm; ^13^C NMR (100 MHz, D_2_O) δ 168.1 (s, 3C, C=O), 143.9 (s, 9C, C-triazole), 135.1 (s, 3C, Ar), 129.0 (d, 3C, Ar), 124.9 (d, 9C, C-triazole), 73.2 (d, 9C, C-4), 67.2 (d, 9C, C-3), 67.1 (d + t, 18C, C-5 + O*CH_2_*CNH), 63.5 (t, 9C, O*CH_2_*-triazole), 60.7 (s, 3C, OCH_2_*C*NH), 56.0 (t, 9C, C-2), 55.3 (t, 9C, C-6), 53.6 (t, 9C, C-1’), 48.2 (t, 9C, C-3’), 25.9 (t, 9C, C-2’) ppm; MS (ESI) *m*/*z* (%): 958.92 (100) [M/3 + Na]^+^; anal. calcd for C_120_H_195_N_39_O_39_: C, 51.33; H, 7.00; N, 19.45; found: C, 51.54; H, 6.79; N, 19.69.

**Protected nonavalent iminosugar 12:** In a similar manner as described in [21] to a solution of **4** (57 mg, 0.22 mmol) in 3 mL of a 2:1 THF/H_2_O mixture, CuSO_4_ (30 mol %, 1.2 mg, 0.007 mmol), sodium ascorbate (60 mol %, 2.9 mg, 0.014 mmol) and **6** (20 mg, 0.024 mmol) were added. The reaction mixture was heated in a MW reactor at 80 °C for 45 min, until TLC analysis showed the disappearance of the starting material. After filtration through Celite^®^, the solvent was removed under reduced pressure and the crude product was purified by gradient FCC (from CH_2_Cl_2_/MeOH 10:1 to CH_2_Cl_2_/MeOH 1:1) and then by size exclusion chromatography, employing Sephadex LH-20^®^ resin and eluting with MeOH, to afford pure **12** (67 mg, 0.021 mmol, 92%) as a pale yellow oil. *R*_f_ 0.29 (CH_2_Cl_2_/MeOH 1:1); [α]_D_^29^ +4.18 (*c* 0.70, CHCl_3_); ^1^H NMR (400 MHz, CDCl_3_) δ 8.14 (s, 3H, Ar), 7.72 (s, 9H, H-triazole), 7.11 (s, 3H, NH), 4.56 (s, 18H, O*CH_2_-*triazole), 4.38 (t, *J* = 6.4 Hz, 18H, 3’-H), 4.24 (q, *J =* 5.4 Hz, 9H, 3-H), 3.96 (t, *J* = 4.9 Hz, 9H, 4-H), 3.89−3.85 (m, 27H, 5-H + O*CH_2_*CNH), 2.60–2.43 (m, 27H, 2-H + 1’-Ha), 2.82–2.51 (m, 27H, 6-H + 1’-Hb), 1.99–1.95 (m, 18H, 2’-H), 1.43 (s, 27H, Me), 1.35 (s, 27H, Me) ppm; ^13^C NMR (100 MHz, CDCl_3_) δ 166.5 (s, 3C, C=O), 144.4 (s, 9C, C-triazole), 135.6 (s, 3C, Ar), 128.6 (d, 3C, Ar), 123.7 (d, 9C, C-triazole), 109,0 (s, 9C, acetal), 77.5 (d, 9C, C-4), 72.3 (d, 9C, C-3), 68.4 (t, 9C, O*CH_2_*CNH), 68.1 (d, 9C, C-5), 64.7 (t, 9C, O*CH_2_*-triazole), 60.6 (s, 3C, OCH_2_*C*NH), 56.1 (t, 9C, C-1’), 54.7 (t, 9C, C-2), 53.4 (t, 9C, C-6), 47.8 (t, 9C, C-3’), 28.3 (q, 9C, Me), 27.2 (t, 9C, C-2’), 26.4 (q, 9C, Me) ppm; MS (ESI) *m*/*z* (%): 1078.53 (100) [M/3 + Na]^+^; IR (CDCl_3_): ν 3346, 2989, 2941, 2823, 1663, 1517, 1467, 1382, 1242, 1090, 1057 cm^−1^; anal. calcd for C_147_H_231_N_39_O_39_: C, 55.72; H, 7.35; N, 17.24; found: C, 55.44; H, 7.69; N, 16.99.

**Hydrochloride salt of polyhydroxylated nonavalent iminosugars 11^.^HCl:** To a solution of **12** (60 mg, 0.019 mmol) in 7 mL of methanol, 0.20 mL of HCl 1 M were added and the mixture was stirred at room temperature for 16 hours, until a TLC analysis attested the disappearance of the starting material. The solvent was removed under reduced pressure and the crude product was purified by size exclusion chromatography, employing Sephadex LH-20^®^ resin and eluting with H_2_O, to afford pure hydrochloride salt of **11** (55 mg, 0.019 mmol, 100%) as a waxy solid; [α]_D_^29^ = −13.50 (*c* 2.30, H_2_O); ^1^H NMR (400 MHz, D_2_O) δ 7.91 (s, 3H, Ar), 7.90 (s, 9H, H-triazole), 4.49 (s, 18H, O*CH_2_*-triazole), 4.36 (t, *J* = 6.6 Hz, 18H, 3’-H), 4.08–4.05 (m, 9H, 3-H), 3.94 (td, *J* = 7.6, 3.7 Hz, 9H, 4-H), 3.79 (s, 18H, O*CH_2_*CNH), 3.61 (br s, 9H, 5-H), 3.25–3.21 (m, 9H), 3.14 (br s, 9H), 3.03–3.00 (m, 27H), 2.81 (br s, 9H), 2.26–2.17 (m, 18H, 2’-H) ppm; ^13^C NMR (100 MHz, D_2_O) δ 168.5 (s, 3C, C=O), 144.0 (s, 9C, C-triazole), 135.2 (s, 3C, Ar), 129.0 (d, 3C, Ar), 125.3 (d, 9C, C-triazole), 70.4 (d, 9C, C-4), 67.3 (d, 9C, O*CH_2_*CNH), 65.4 (d, 9C, C-3), 64.9 (t, 9C, C-5), 63.4 (t, 9C, O*CH_2_*-triazole), 60.7 (s, 3C, OCH_2_*C*NH), 53.7 (t, 27C, C-1’ + C-2 + C-6), 47.3 (t, 9C, C-3’), 24.4 (t, 9C, C-2’) ppm; MS (ESI) *m*/*z* (%): 936.57 (100) [M/3 + H]^+^.

**Protected monovalent iminosugar 14:** Analogously as described in [21] to a solution of **4** (49 mg, 0.190 mmol) in 3 mL of a 2:1 THF/H_2_O mixture, CuSO_4_ (30 mol %, 9.1 mg, 0.057 mmol), sodium ascorbate (60 mol %, 23 mg, 0.114 mmol) and **13** (12 mg, 0.220 mmol) were added. The reaction mixture was stirred at room temperature for 16 h, until TLC analysis showed the disappearance of the starting material. After filtration through Celite^®^, the solvent was removed under reduced pressure and the crude product was purified by gradient FCC (from CH_2_Cl_2_/MeOH 20:1 to CH_2_Cl_2_/MeOH/NH_4_OH 6% 10:1:0.2) affording pure **14** (57 mg, 0.183 mmol, 96%) as a pale yellow oil. *R*_f_ 0.88 (CH_2_Cl_2_/MeOH/NH_4_OH 6% 10:1:0.2); [α]_D_^28^ −13.36 (*c* 1.25, MeOH); ^1^H NMR (400 MHz, CD_3_OD) δ 7.91 (s, 1H, H-triazole), 4.48–4.42 (m, 2H, 3’-H), 4.28 (dd, *J =* 8.8, 3.9 Hz, 1H, 3-H), 3.93 (s, 2H, *CH_2_*NH_2_), 3.86–3.78 (m, 2H, 4-H + 5-H), 2.87 (dd, *J* = 12.2, 3.1 Hz, 1H, 2-Ha), 2.70–2.64 (m, 1H, 6-Ha), 2.51–2.43 (m, 1H, 2-Hb), 2.40–2.30 (m, 2H, 1’-H), 2.11–2.03 (m, 3H, 6-Hb + 2’-H), 1.48 (s, 3H, Me), 1.33 (s, 3H, Me) ppm; ^13^C NMR (100 MHz, CD_3_OD) δ 146.8 (s, C-triazole), 122.6 (d, C-triazole), 108.7 (s, acetal), 78.7 (d, C-4), 73.1 (d, C-3), 69.0 (d, C-5), 56.0 (t, C-6), 53.7 (t, C-2), 53.5 (t, C-1’), 47.7 (t, C-3’), 35.8 (t, CH_2_NH_2_), 27.2 (q, Me), 26.9 (t, C-2’), 25.2 (q, Me) ppm; MS (ESI) *m*/*z* (%): 312.17 (100) [M + H]^+^; IR (MeOH): ν 3419, 3305, 3146, 2994, 2808, 1469, 1422, 1381, 1106, 1062 cm^−1^; anal. calcd for C_14_H_25_N_5_O_3_: C, 54.00; H, 8.09; N, 22.49; found: C, 55.88; H, 8.15; N, 22.81.

**Deprotected monovalent iminosugar 15:** In a similar manner as described in [25] a solution of **14** (24 mg, 0.077 mmol) in MeOH (3 mL) was left stirring with 1 M HCl (7 drops) at room temperature for 16 h. The crude mixture was concentrated to yield the hydrochloride salt of **15**. The corresponding free amine was obtained by passing the hydrochloride salt through DOWEX 50XW8-100 ion-exchange resin. Elution with 6% NH_4_OH afforded the free base **15** (16 mg, 0.059 mmol, 77%). [α]_D_^24^ −11.43 (*c* 0.70, MeOH); ^1^H NMR (400 MHz, CD_3_OD) δ 7.91 (s, 1H, H-triazole), 4.42–4.34 (m, 2H, 3’-H), 3.97 (s, 2H, *CH_2_*NH_2_), 3.80 (dt, *J =* 5.7, 2.9 Hz, 1H, 3-H), 3.68 (td, *J* = 7.9, 4.1 Hz, 1H, 4-H), 3.30 (d, *J* = 4.4 Hz, 1H, 5-H), 2.70−2.59 (m, 2H, 2-Ha + 6-Ha), 2.31–2.17 (m, 3H, 2-Hb + 1’-H), 2.03–1.93 (m, 3H, 6-Hb + 2’-H) ppm; ^13^C NMR (50 MHz, CD_3_OD) δ 144.1 (s, C-triazole), 123.3 (d, C-triazole), 73.9 (d, C-4), 68.3 (d, C-5), 67.9 (d, C-3), 56.5 (t, C-6), 56.1 (t, C-2), 53.7 (t, C-1’), 47.9 (t, C-3’), 35.2 (t, CH_2_NH_2_), 26.9 (t, C-2’) ppm; MS (ESI) *m*/*z* (%): 293.87 (100) [M + Na]^+^; anal. calcd for C_11_H_21_N_5_O_3_: C, 48.70; H, 7.80; N, 25.81; found: C, 48.49; H, 7.71; N, 26.19.

## Supporting Information

File 1 Characterization data, ^1^H NMR and ^13^C NMR spectra of synthesized compounds and IC_50_ graphics of compounds **11**·HCl and **15**.
