# Supplementary material for: Exploring architectures displaying multimeric presentations of a trihydroxypiperidine iminosugar
Source: Beilstein J Org Chem. 2015 Dec 16;11:2631–40. doi: 10.3762/bjoc.11.282 (PMC4685925; doi:10.3762/bjoc.11.282)
Supplement: File 1 — Characterization data, 1H NMR and 13C NMR spectra of synthesized compounds and IC50 graphics of compounds 11·HCl and 15. [file Beilstein_J_Org_Chem-11-2631-s001.pdf]

Supporting Information

for

Exploring architectures displaying multimeric presentations of a trihydroxypiperidine iminosugar

Camilla Matassini[1], Stefania Mirabella[1], Andrea Goti[1], Inmaculada Robina[2], Antonio J. Moreno-Vargas[2] and Francesca Cardona*,[1]

Address: [1]Dipartimento di Chimica "Ugo Schiff", Università di Firenze, Via della Lastruccia 3-13, 50019 Sesto Fiorentino (FI), Italy and [2]Departamento de Química Orgánica, Facultad de Química, Universidad de Sevilla, c/ Prof. García González 1, E-41012, Sevilla, Spain.

Email: Francesca Cardona - francesca.cardona@unifi.it

*Corresponding author

Characterization data, ^{1}H NMR and ^{13}C NMR spectra of synthesized compounds and IC_{50} graphics of compounds 11·HCl and 15

Table of contents

Figure S1. ^{1}H NMR spectrum of compound **7** S3

Figure S2. ^{13}C NMR spectra of compound **7** S4

Figure S3. ^{1}H NMR spectrum of compound **9** S5

Figure S4. ^{13}C NMR spectra of compound **9** S6

Figure S5. ^{1}H NMR spectrum of compound **8** S7

Figure S6. ^{13}C NMR spectra of compound **8** S8

Figure S7. ^{1}H NMR spectrum of compound **11** S9

Figure S8. ^{13}C NMR spectra of compound **11** S10

Figure S9. ^{1}H NMR spectrum of compound **12** S11

Figure S10. ^{13}C NMR spectra of compound **12** S12

Figure S11. ^{1}H NMR spectrum of compound **11·HCl** S13

Figure S12. ^{13}C NMR spectra of compound **11·HCl** S14

Figure S13. ^{1}H NMR spectrum of compound **14** S15

Figure S14. ^{13}C NMR spectra of compound **14** S16

Figure S15. ^{1}H NMR spectrum of compound **15** S17

Figure S16. ^{13}C NMR spectra of compound **15** S18

Figure S17. IC_{50} of compound **11** towards amyloglucosidase. S19

Figure S18. IC_{50} of compound **15** towards amyloglucosidase. S19

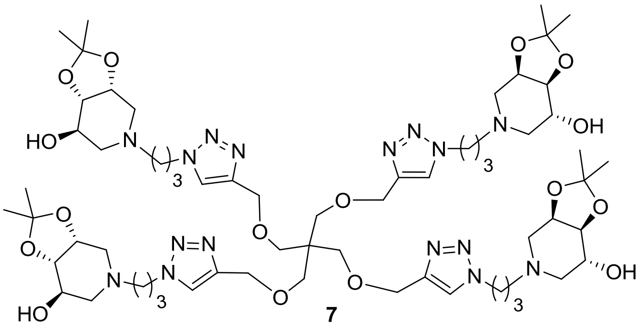

7

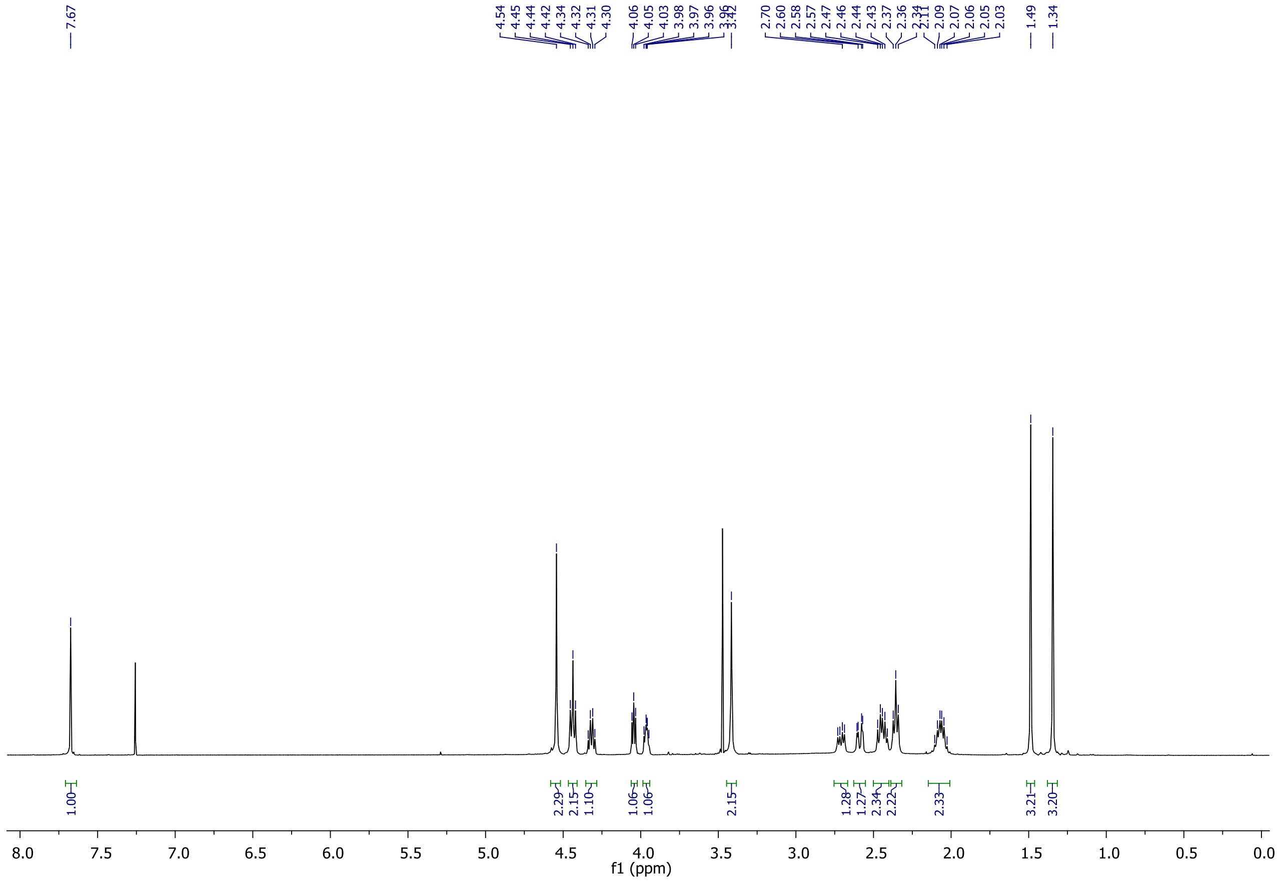

Figure S1: ^{1}H NMR spectrum of compound **7** (400 MHz, $CDCl_3$).

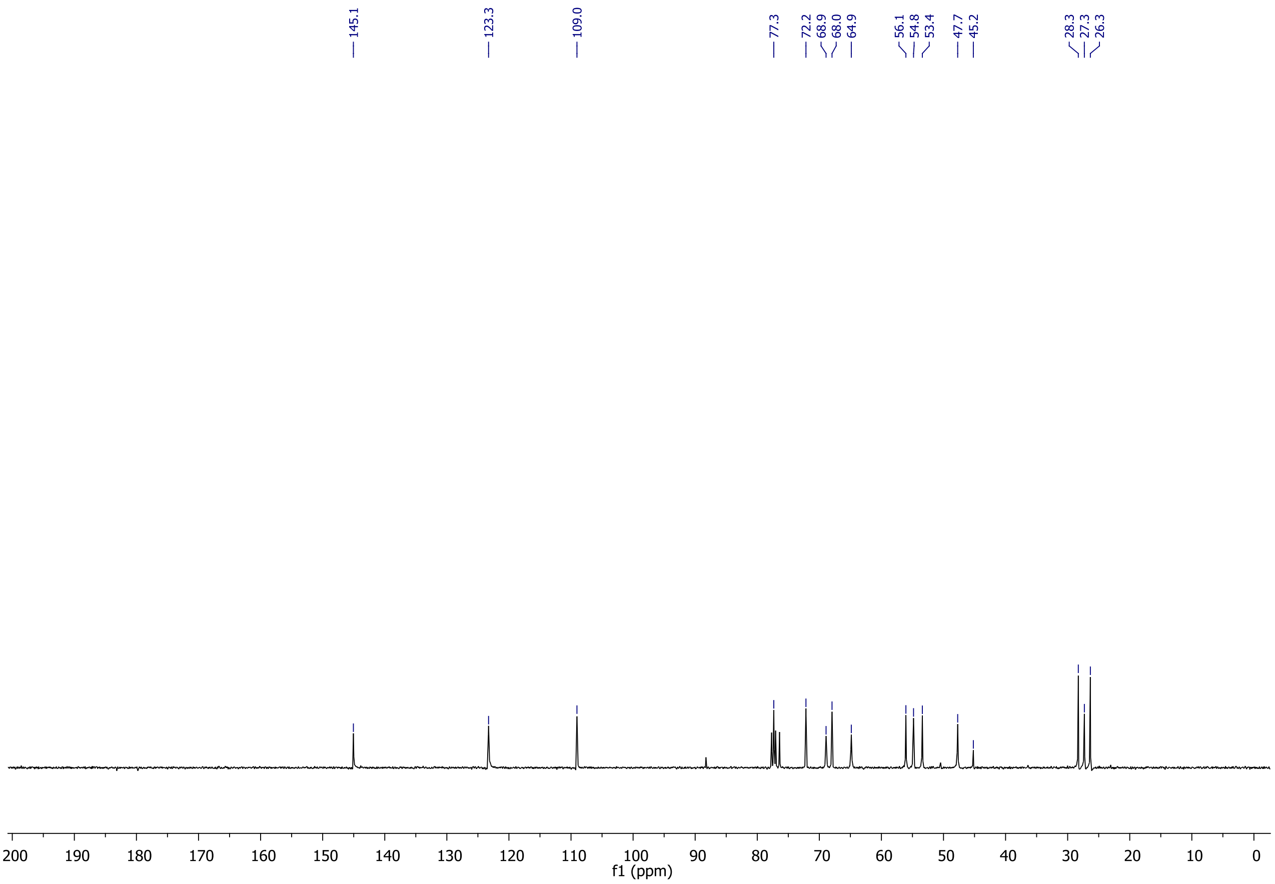

Figure S2: ^{13}C NMR spectrum of compound **7** (50 MHz, $CDCl_3$).

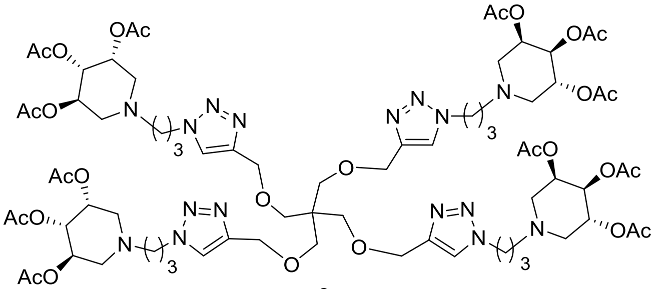

9

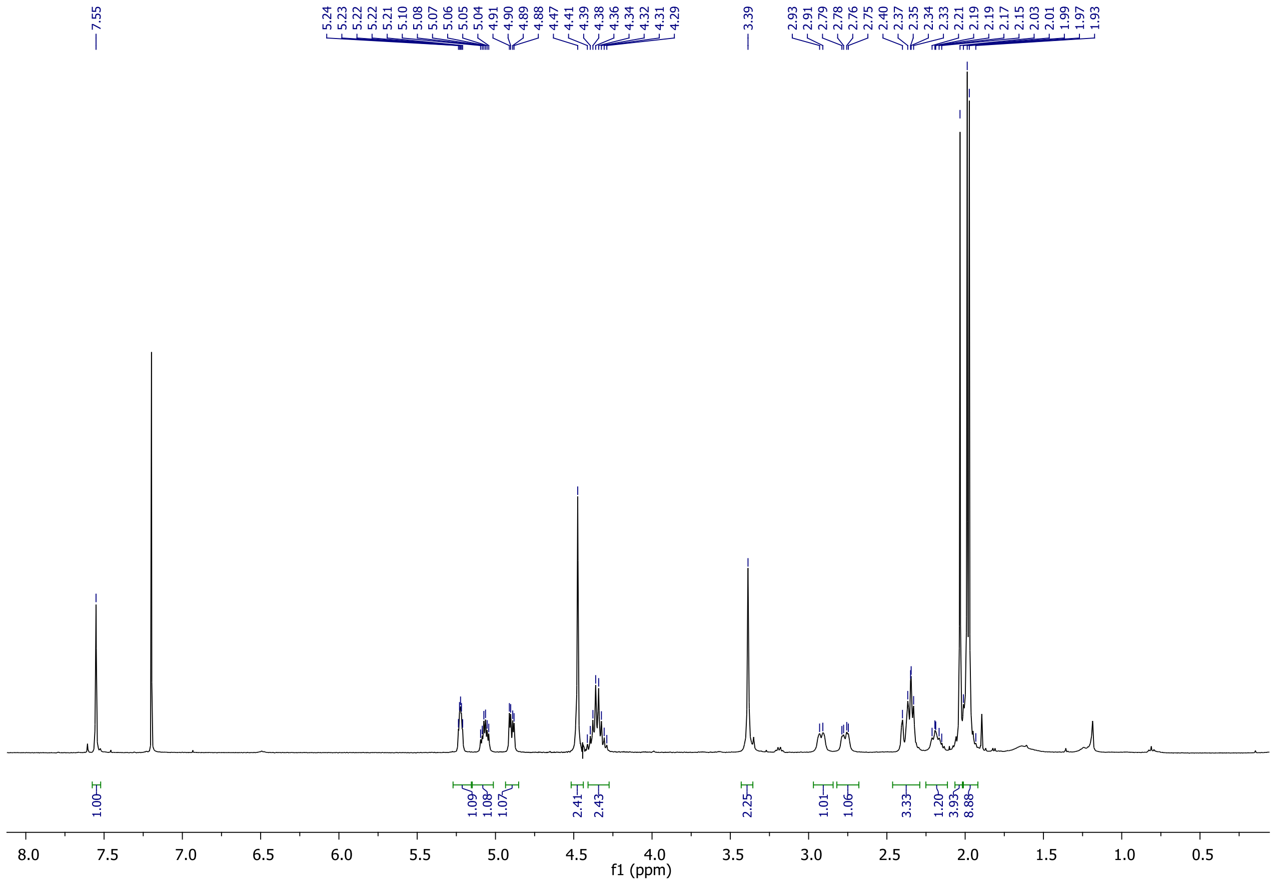

Figure S3: ^{1}H NMR spectrum of compound **9** (400 MHz, $CDCl_3$).

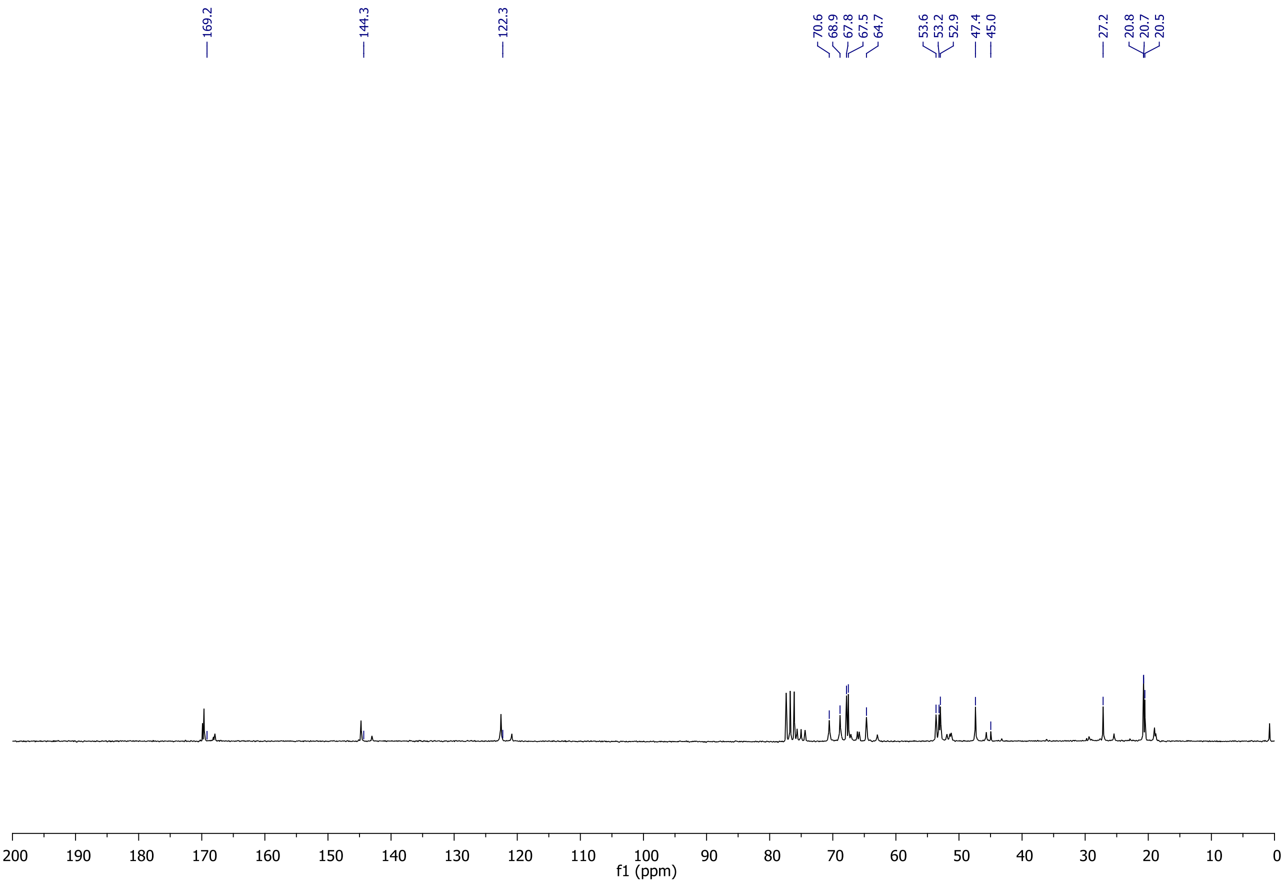

Figure S4: ^{13}C NMR spectrum of compound **9** (50 MHz, $CDCl_3$).

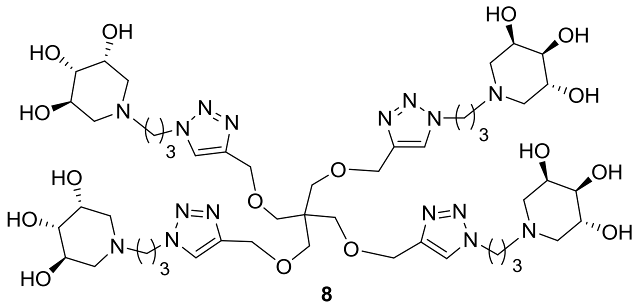

8

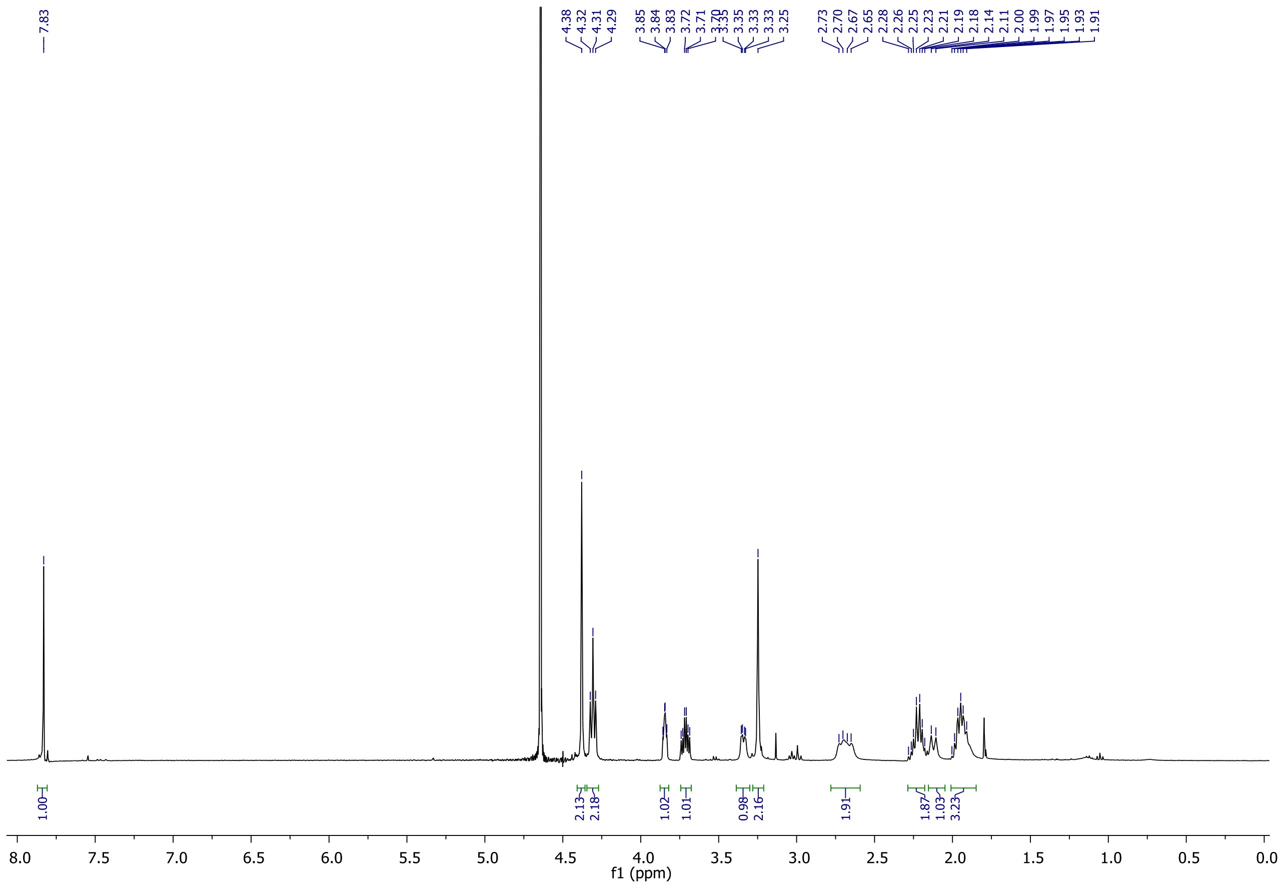

Figure S5: ^{1}H NMR spectrum of compound **8** (400 MHz, D_2O).

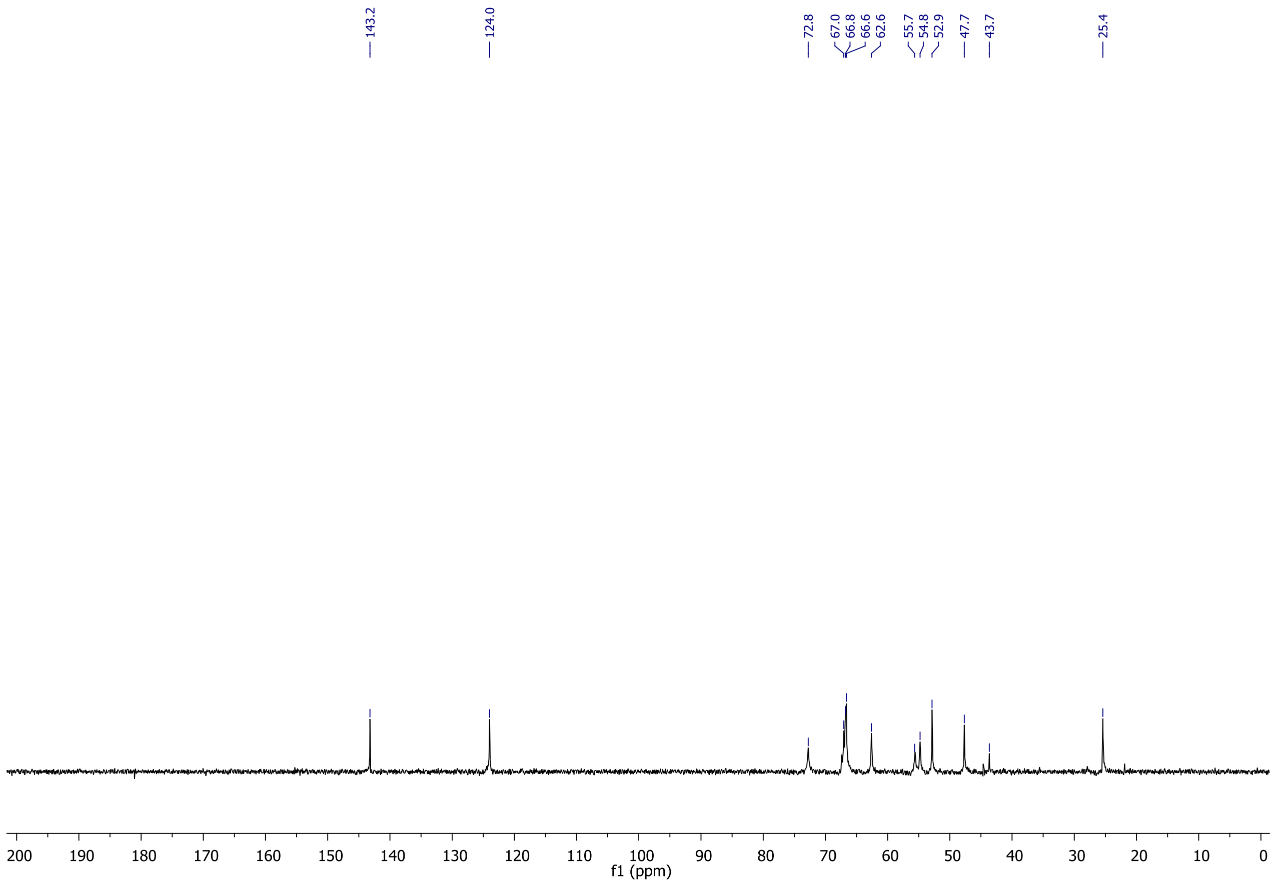

Figure S6: ^{13}C NMR spectrum of compound **8** (50 MHz, D_2O).

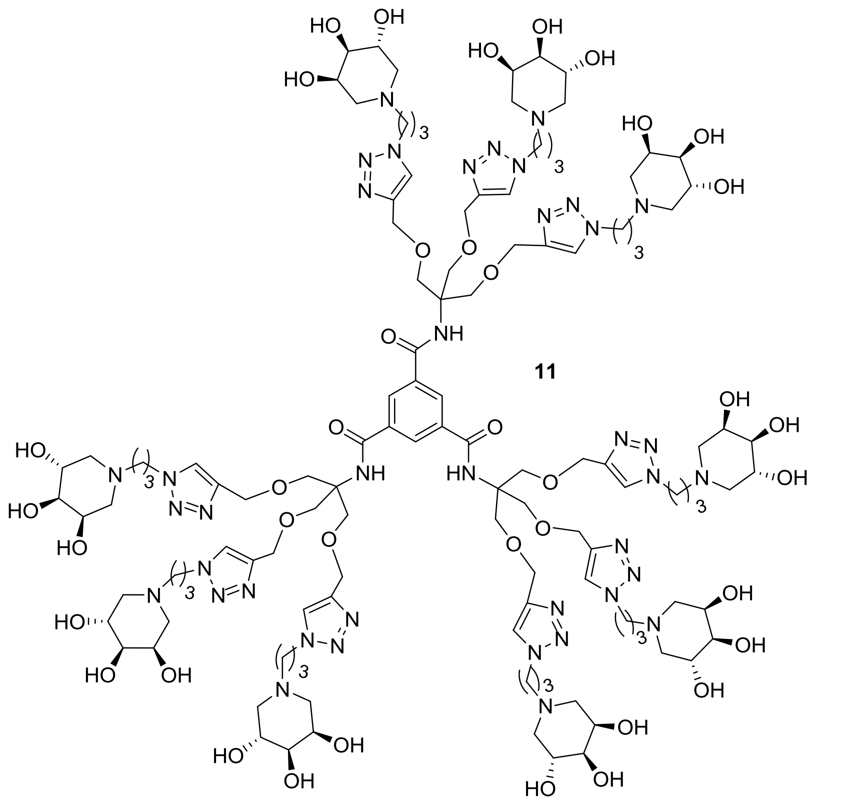

11

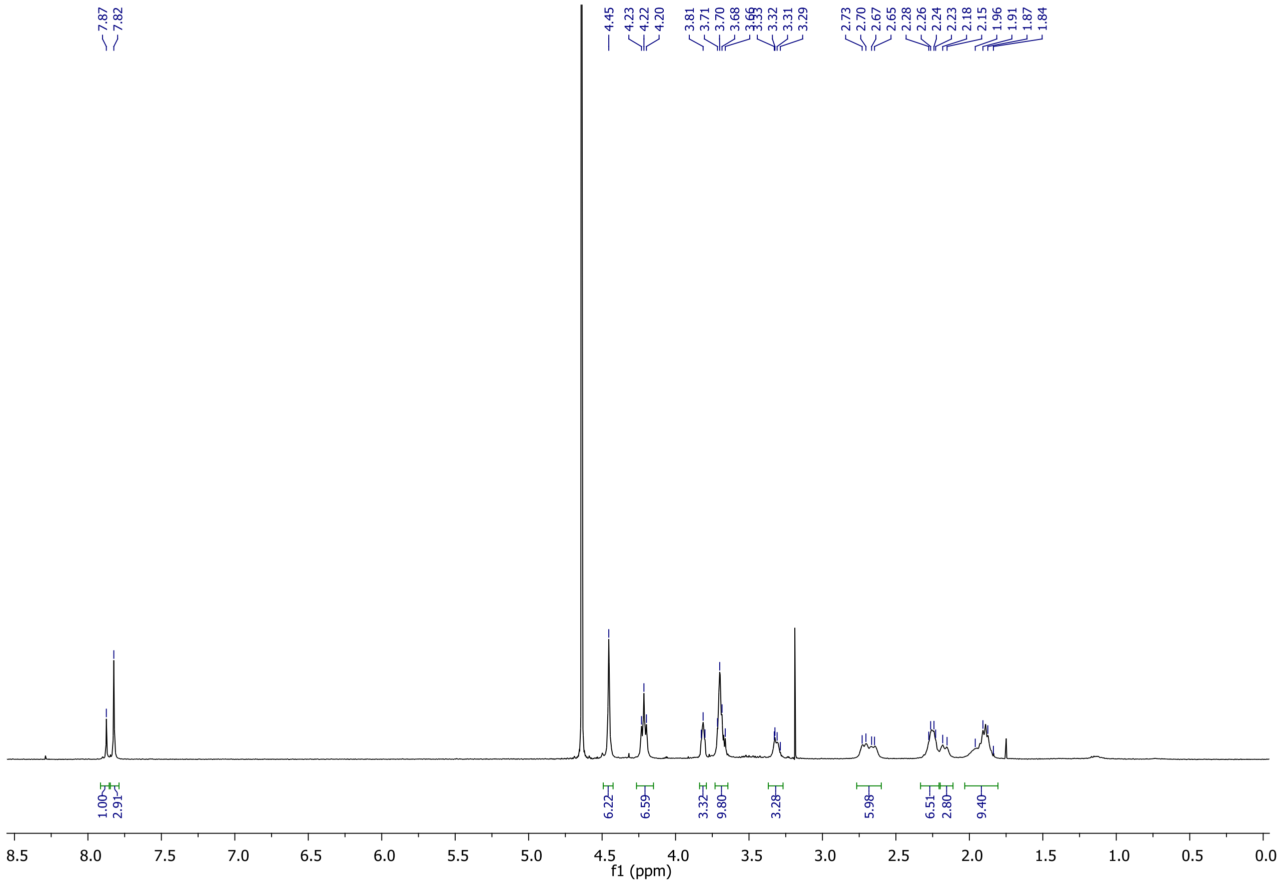

Figure S7: ^{1}H NMR spectrum of compound **11** (400 MHz, D_2O).

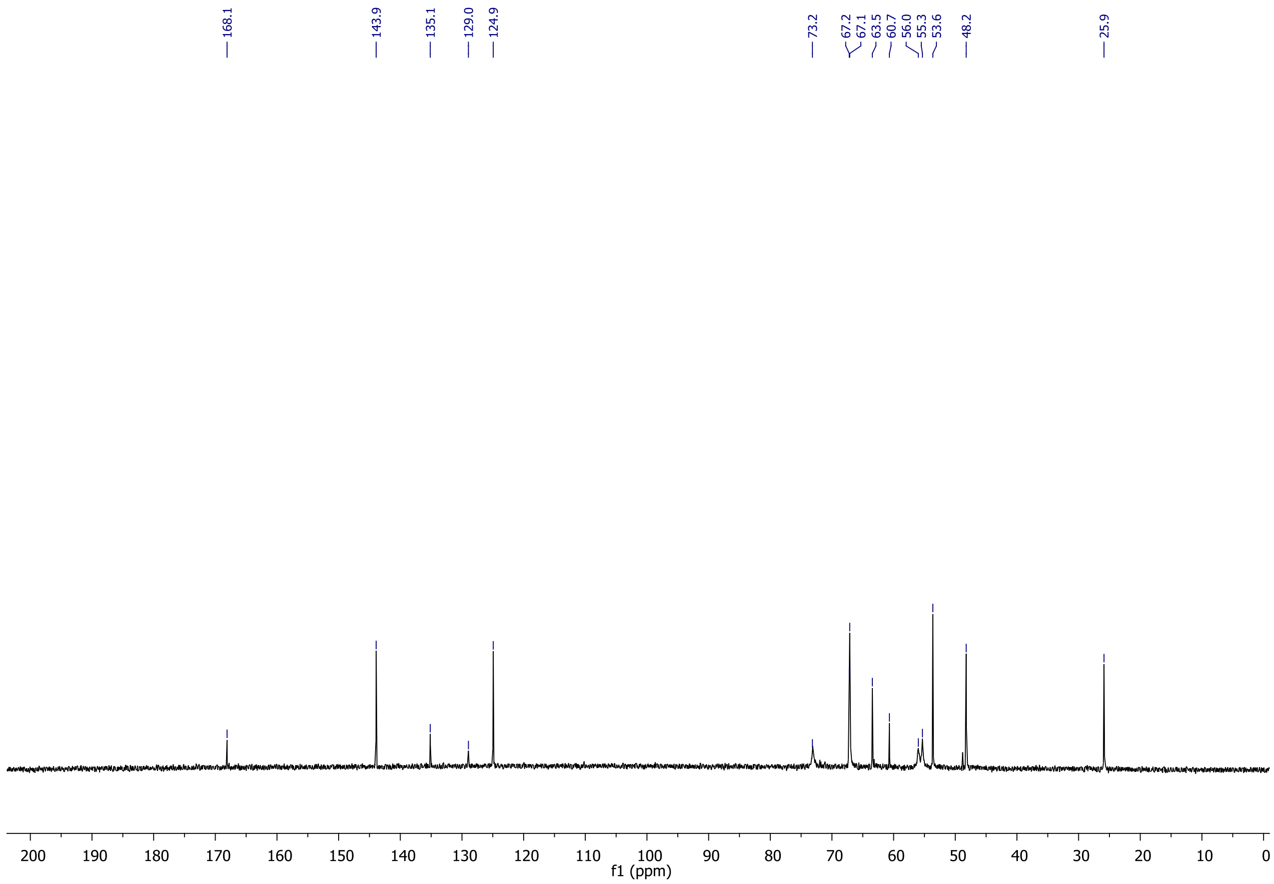

Figure S8: ^{13}C NMR spectrum of compound **11** (100 MHz, D_2O).

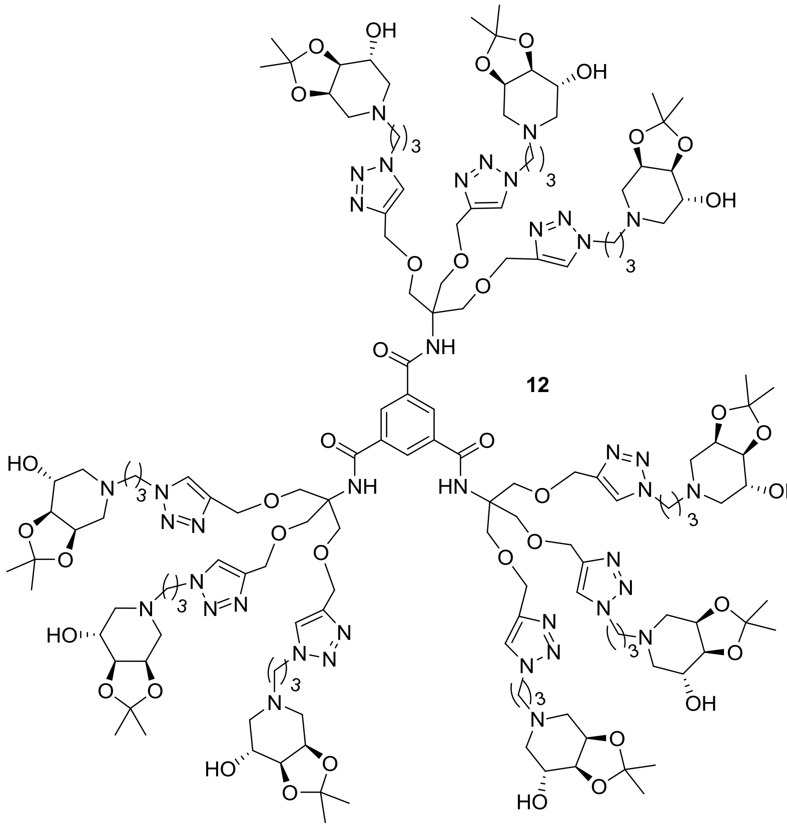

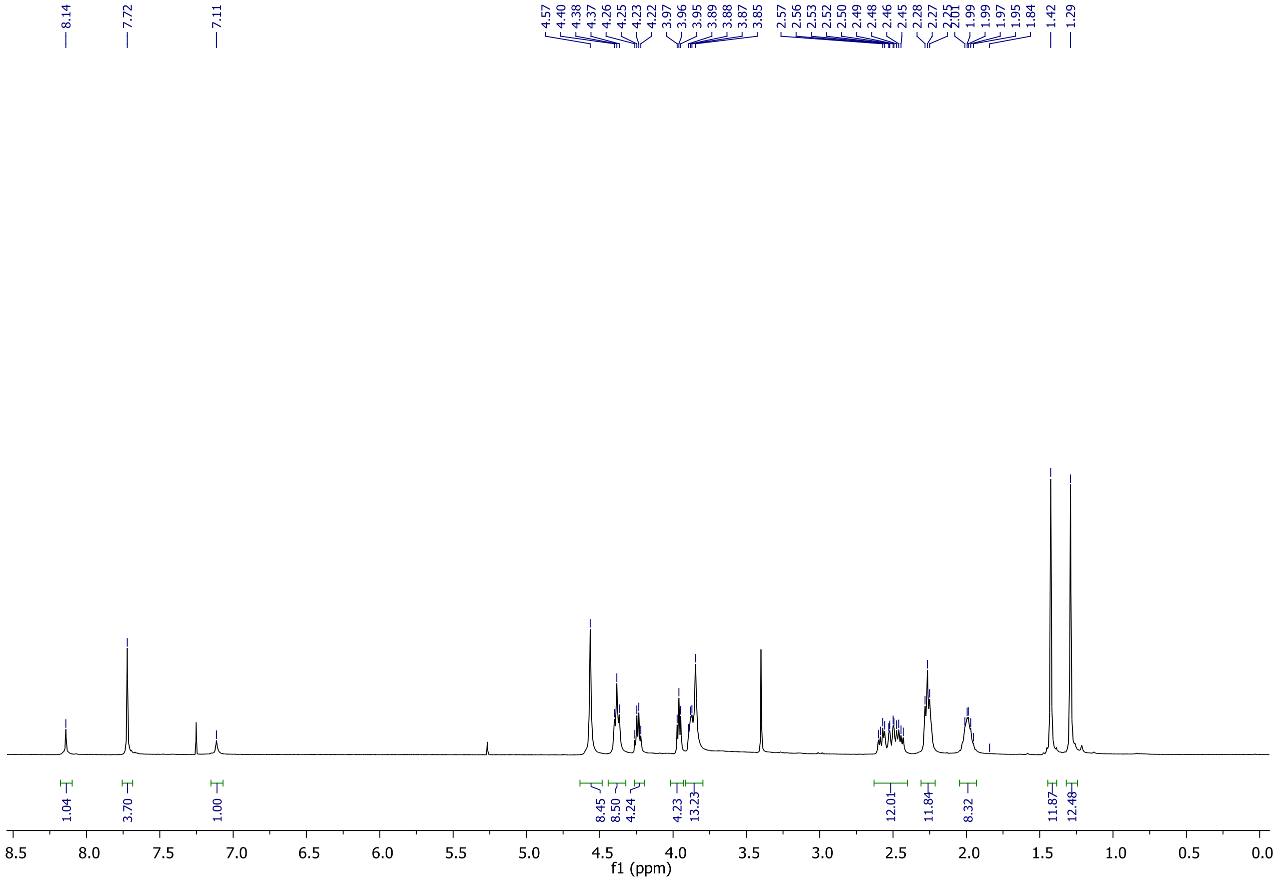

Figure S9: ^{1}H NMR spectrum of compound **12** (400 MHz, $CDCl_3$).

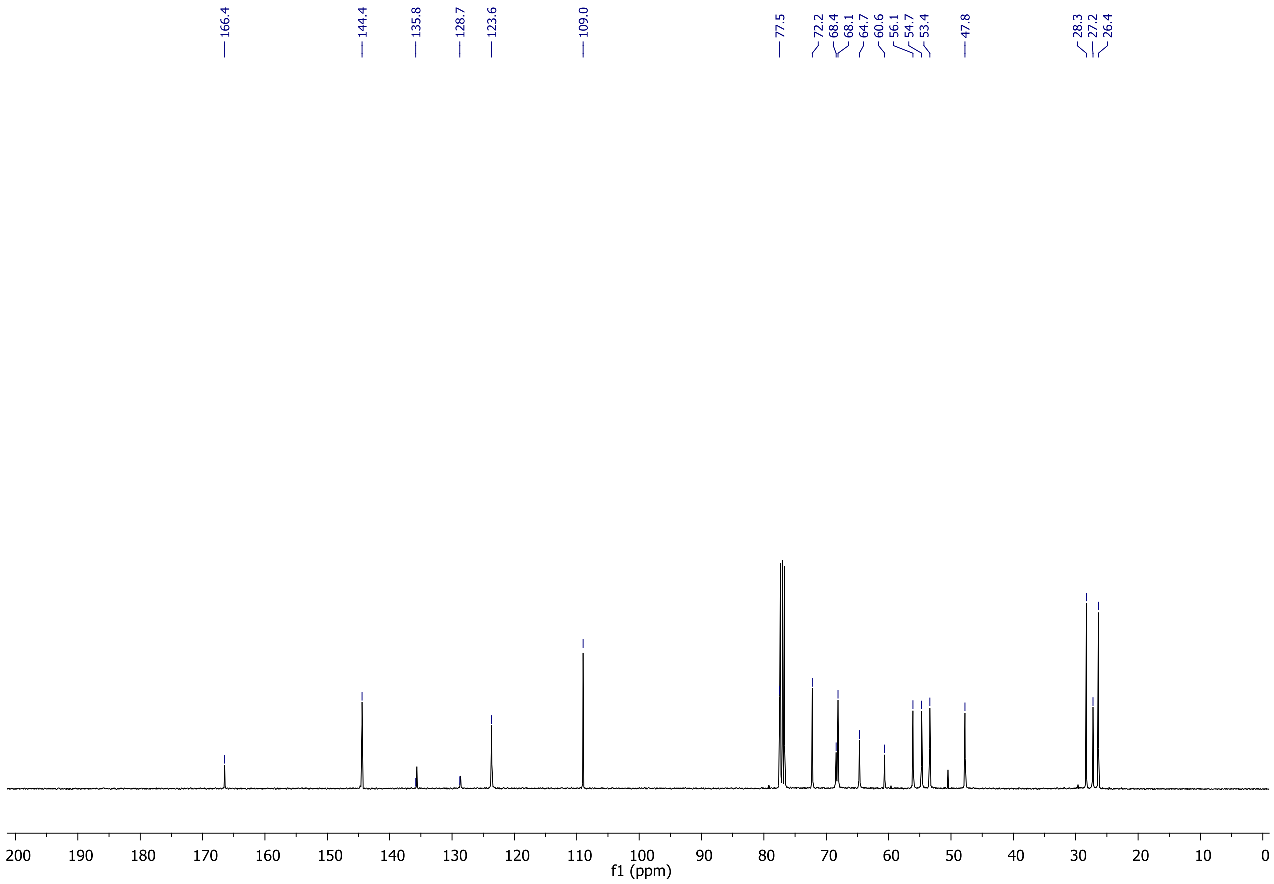

Figure S10: ^{13}C NMR spectrum of compound **12** (100 MHz, $CDCl_3$).

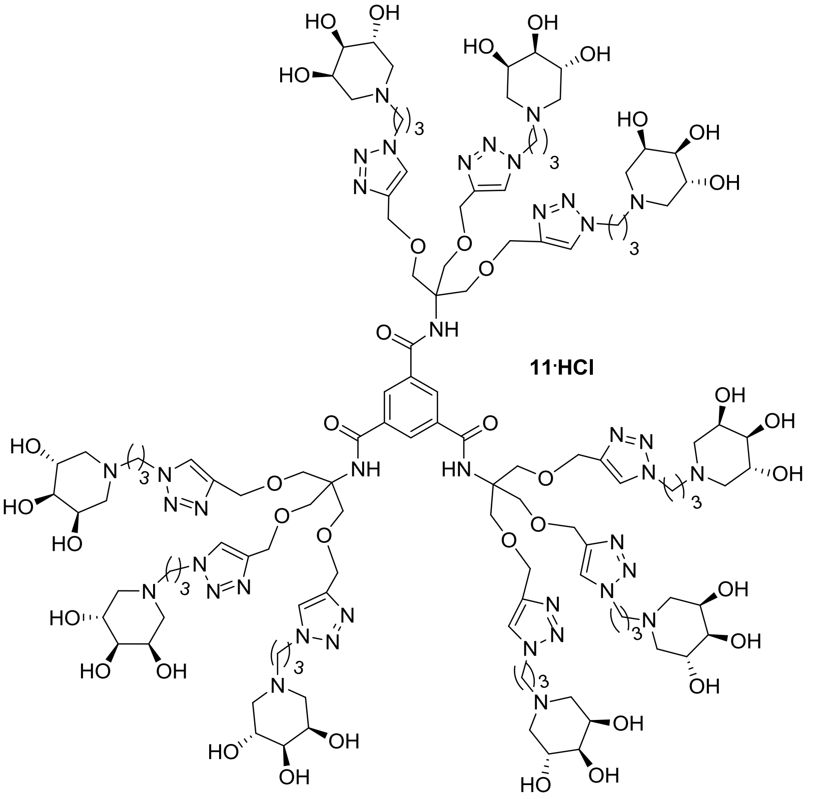

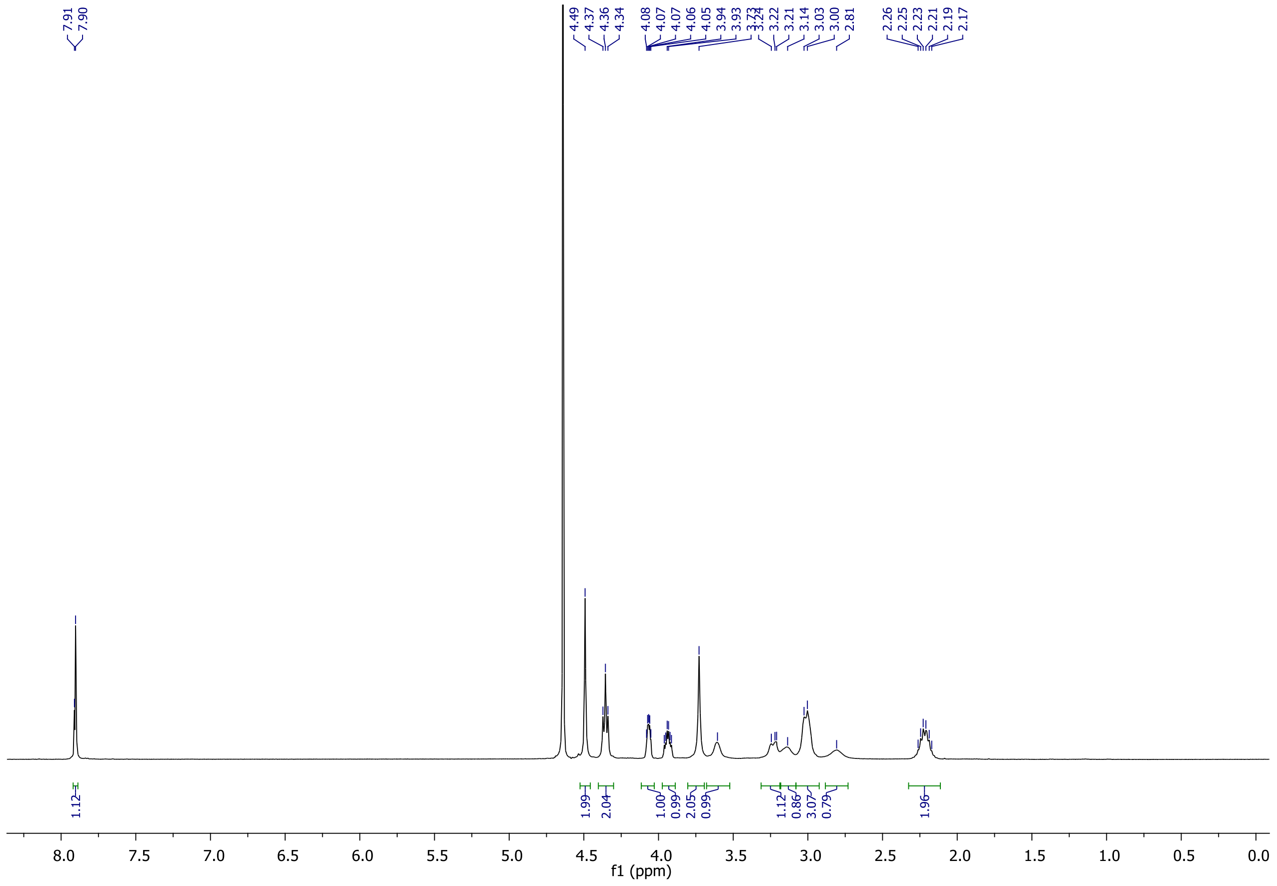

Figure S11: ^{1}H NMR spectrum of compound **11**·HCl (400 MHz, D_2O).

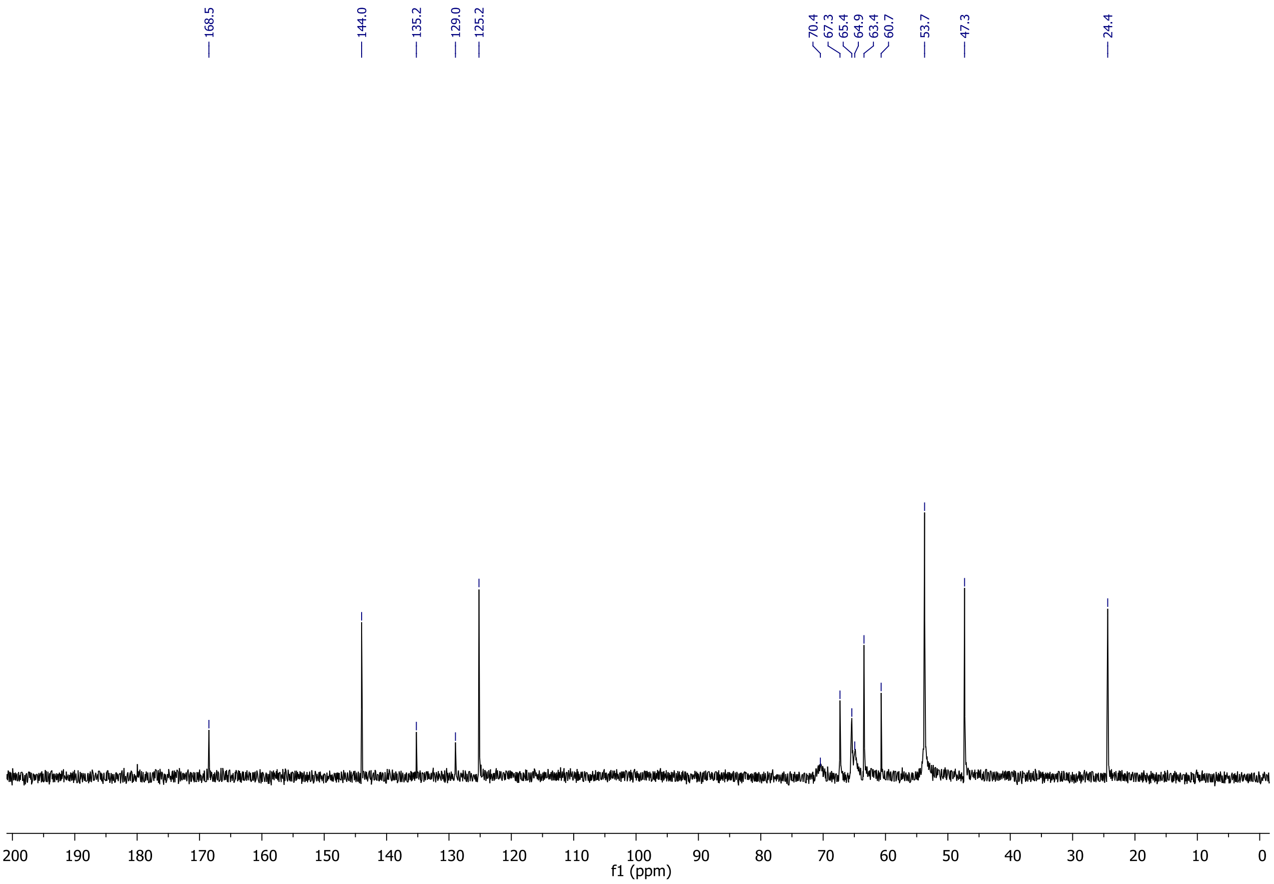

Figure S12: ^{13}C NMR spectrum of compound **11**·HCl (100 MHz, D_2O).

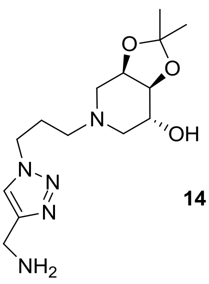

14

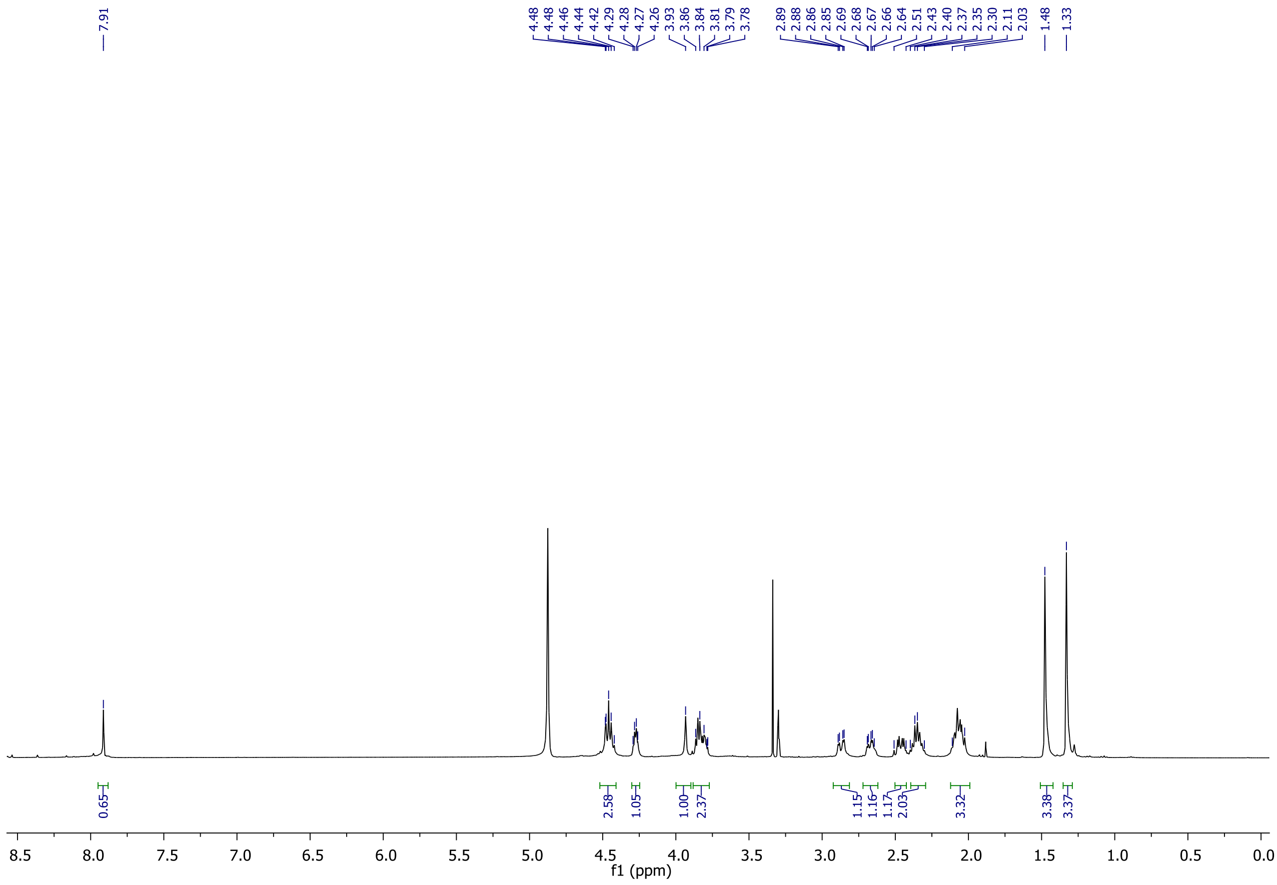

Figure S13: ^{1}H NMR spectrum of compound **14** (400 MHz, CD_3OD).

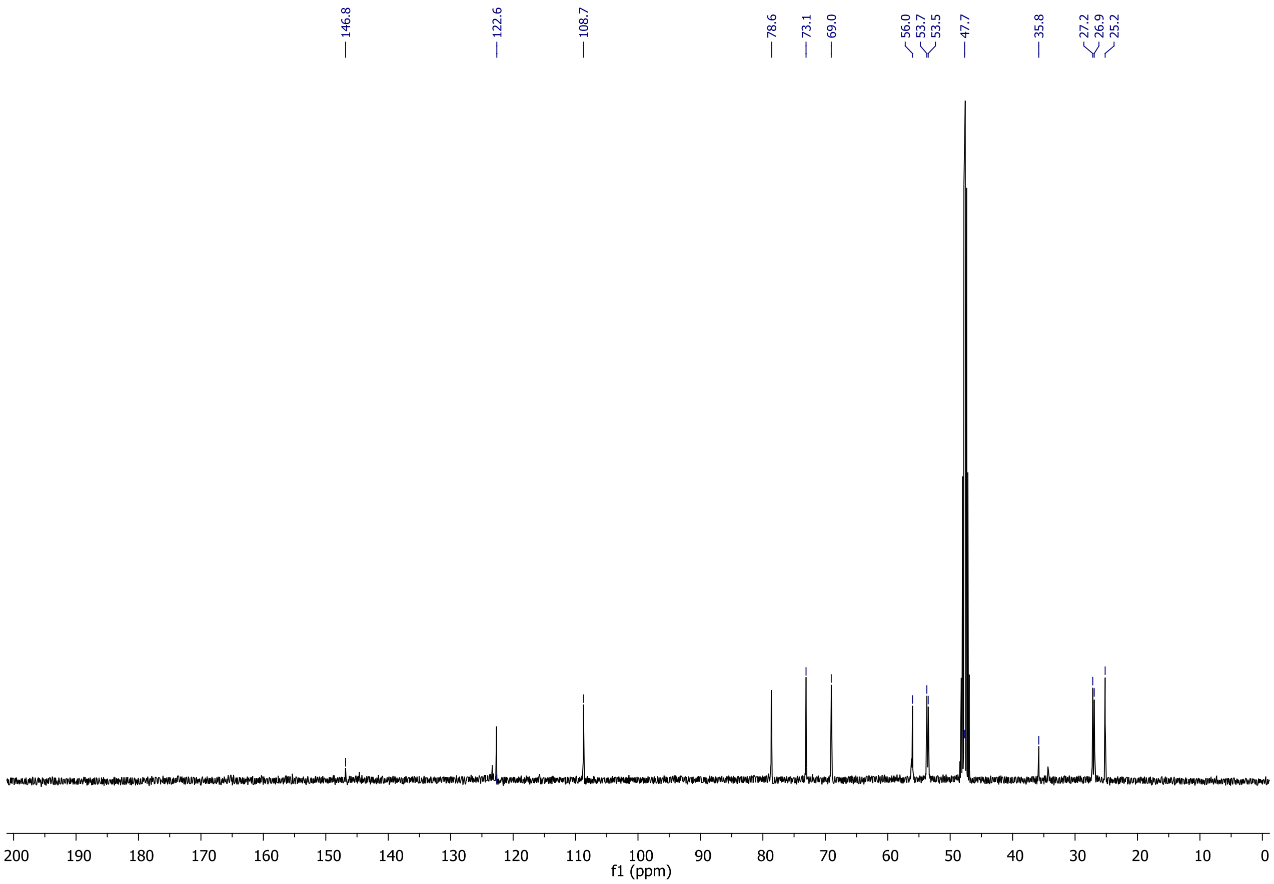

Figure S14: ^{13}C NMR spectrum of compound **14** (100 MHz, CD_3OD).

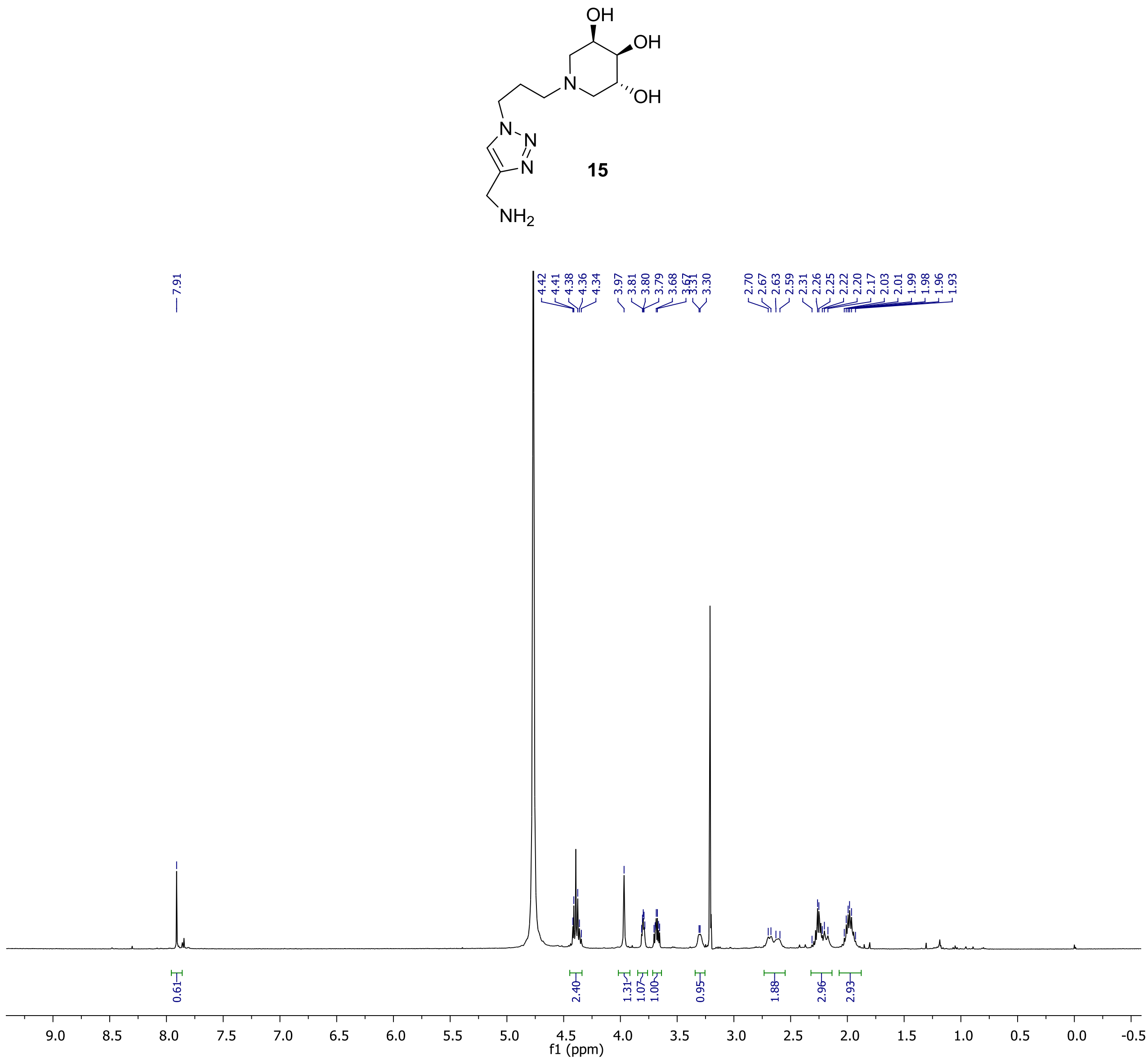

Figure S15: ^{1}H NMR spectrum of compound **15** (400 MHz, CD_3OD).

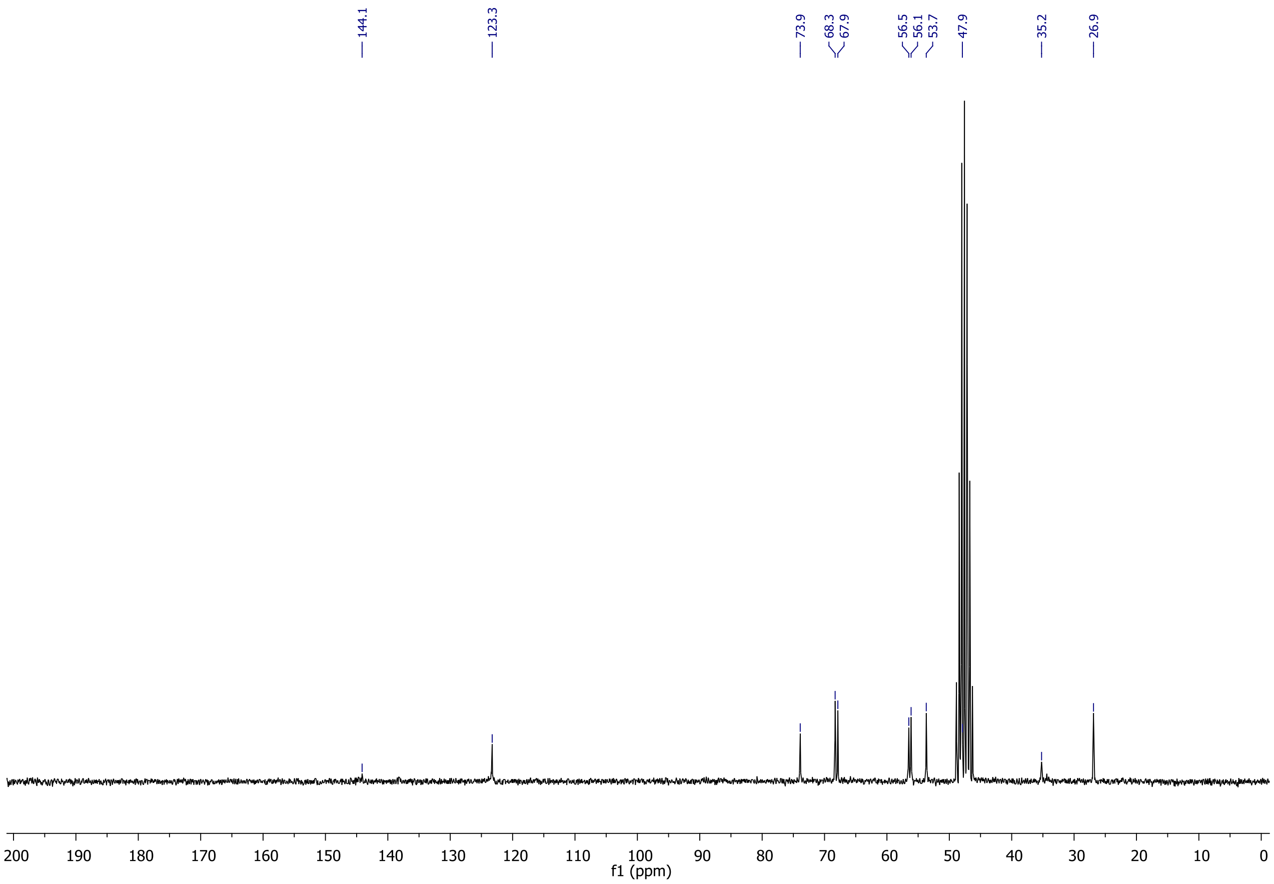

Figure S16: ^{13}C NMR spectrum of compound **15** (50 MHz, CD_3OD).

Glycosidase inhibition assays

The experiments were performed essentially as previously described.[1] Briefly, 0.01–0.5 units/mL of enzyme and inhibitor were pre-incubated for 5 min at rt, and the reaction started by addition of the substrate, buffered to the optimal pH of the enzyme. After 20 min of incubation at 37 °C, the reaction was stopped by addition of sodium borate buffer pH 9.8. The *p*-nitrophenolate formed was measured by visible absorption spectroscopy at 405 nm.

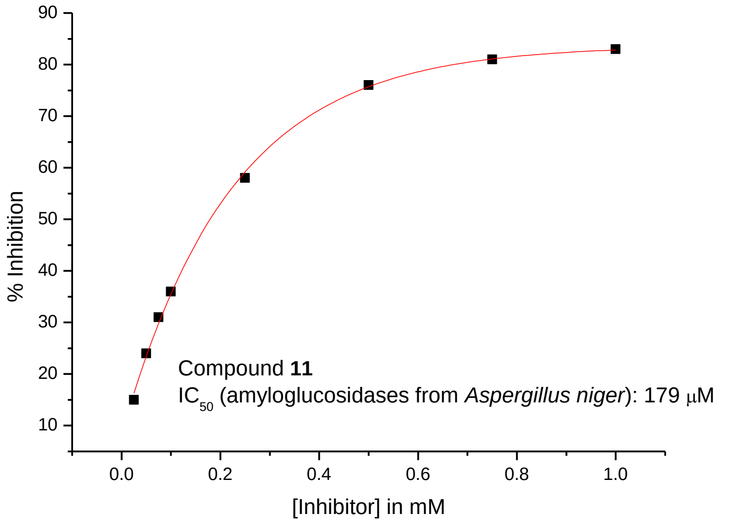

Figure S17: IC_{50} of compound **11** towards amyloglucosidase.

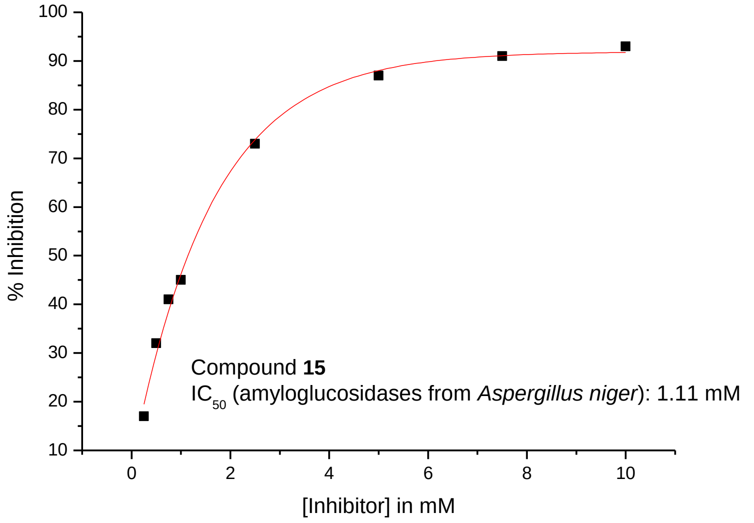

Figure S18: IC_{50} of compound **15** towards amyloglucosidase.

[1] (a) Saul, R.; Chambers, J. P.; Molyneux, R. J.; Elbein, A. D. *Arch. Biochem. Biophys.* **1983**, *221*, 593–597; (b) Brandi, A.; Cicchi, S.; Cordero, F. M.; Frignoli, R.; Goti, A.; Picasso, S.; Vogel, P. *J. Org. Chem.* **1995**, *60*, 6806–6812.
